# Enriching and Quantifying Porous Single Layer 2D Polymers by Exfoliation of Chemically Modified van der Waals Crystals

**DOI:** 10.1002/anie.201912705

**Published:** 2020-01-23

**Authors:** Ralph Z. Lange, Kevin Synnatschke, Haoyuan Qi, Niklas Huber, Gregor Hofer, Baokun Liang, Christian Huck, Annemarie Pucci, Ute Kaiser, Claudia Backes, A. Dieter Schlüter

**Affiliations:** ^1^ Institute for Polymers ETH Zürich Vladimir-Prelog-Weg 5 8093 Zürich Switzerland; ^2^ Institute of Physical Chemistry Heidelberg University Im Neuenheimer Feld 253 69120 Heidelberg Germany; ^3^ Central Facility of Electron Microscopy Electron Microscopy Group of Materials Science Ulm University Albert-Einstein-Allee 11 89081 Ulm Germany; ^4^ X-ray Platform D-MATL Department of Materials ETH Zürich Vladimir-Prelog-Weg 5 8093 Zürich Switzerland; ^5^ Kirchhoff Institute of Physics Heidelberg University Im Neuenheimer Feld 227 69120 Heidelberg Germany

**Keywords:** layered compounds, liquid phase exfoliation, monolayers, polymers, post-polymerization modification

## Abstract

2D polymer sheets with six positively charged pyrylium groups at each pore edge in a stacked single crystal can be transformed into a 2D polymer with six pyridines per pore by exposure to gaseous ammonia. This reaction furnishes still a crystalline material with tunable protonation degree at regular nano‐sized pores promising as separation membrane. The exfoliation is compared for both 2D polymers with the latter being superior. Its liquid phase exfoliation yields nanosheet dispersions, which can be size‐selected using centrifugation cascades. Monolayer contents of ≈30 % are achieved with ≈130 nm sized sheets in mg quantities, corresponding to tens of trillions of monolayers. Quantification of nanosheet sizes, layer number and mass shows that this exfoliation is comparable to graphite. Thus, we expect that recent advances in exfoliation of graphite or inorganic crystals (e.g. scale‐up, printing etc.) can be directly applied to this 2D polymer as well as to covalent organic frameworks.

## Graphical abstract

**A 2D polymer** with pyrylium groups is transformed into a 2D polymer with pyridine groups by exposure to gaseous ammonia. Liquid phase exfoliation of the latter yields nanosheet dispersions, which can be size‐selected using centrifugation cascades. Nanosheet quantification shows that the exfoliation is comparable to graphite, demonstrating the promising potential of this approach, which could also be applied to covalent organic frameworks.

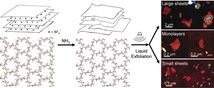

## Introduction

**Figure 1:**
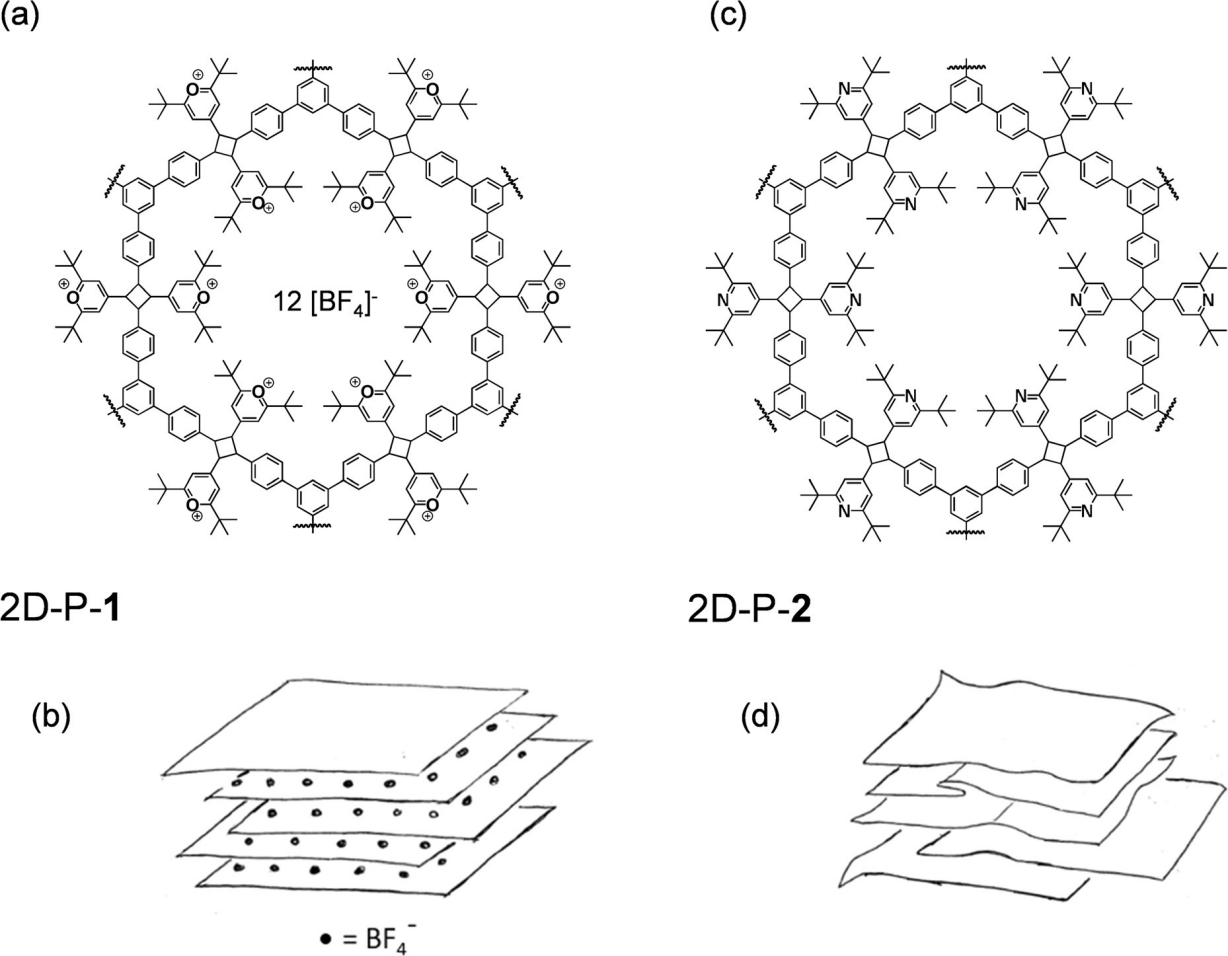
Structure of the 2D polymers under investigation. a,c) Section of chemical structures of 2D‐P‐**1** and 2D‐P‐**2** showing the differences in pore edge decoration with the pyrylium ions in 2D‐P‐**1** and the pyridines in 2D‐P‐**2** (the wiggly lines indicate where the network structures continue in fully ordered fashion). b,d) Layered structures of 2D‐P‐**1** and 2D‐P‐**2**. The packing of 2D‐P‐**1** contains positively charged organic layers fully separated from one another by a layer of counter ions as the sketch suggests and as is observed in some inorganic 2D materials. Substituents at the pore edges can mutually interdigitate such that a tight arrangement results with the counter ions in between. The packing of 2D‐P‐**2** while maintaining some residual order, local stresses during post‐polymerization modification are expected to cause sheets to stretch, compress, shift and bend. The formation of folds, as shown, is speculative.

The structure of the 2D polymer^1^ **1** (2D‐P‐**1**, Figure 1 a,b) has been established by single crystal X‐ray diffraction (sc‐XRD).^2^ It comes as ABC stacks of laterally “infinitely” extended long range ordered, porous, covalent sheets, which expose six positively charged pyrylium tetrafluoroborate groups into each of its 1–2 nm sized hexagonal pores. Initial wet exfoliation experiments of these crystals afforded monolayer sheets of 2D‐P‐**1**. However, these were rare events and a fraction with a large and quantifiable number of monolayers could never be provided.^2^ Covalently bonded, strong and large all‐carbon sheets such as 2D‐P‐**1** are attractive for applications as novel membrane materials for gas and ion separation.^3^ This explains the strong need for a substantially improved exfoliation process that provides monolayer‐rich and quantifiable fractions of 2D polymers, which initially are often obtained in single crystalline stacked form.1a, 4

Recently, liquid phase exfoliation (LPE) under tip‐sonication combined with liquid cascade centrifugation (LCC)^5^ has greatly improved the accessibility of inorganic layered 2D materials down to monolayers in the liquid phase. It was an obvious goal to apply this method to 2D polymers as well. As charged systems may not be ideally suited for this method,^6^ we wondered whether 2D polymer 2D‐P‐**1** could be converted into a neutral derivative like 2D‐P‐**2** (Figure 1 c) prior to exfoliation. This would have the benefit of a direct comparability between these two structurally closely related cases: 2D polymers **1** and **2** just differ in pore edge chemistry and charge state while showing the same network structure. Additionally, it would be attractive because of the excellent availability of polymer 2D‐P‐**1**,^2^ of which single crystals are accessible on a multi‐gram scale at little effort. For this conversion, the well‐established and extremely high yielding reaction of pyrylium ions into pyridines in the presence of ammonia gas^7^ appeared attractive. All pyrylium ions of 2D‐P‐**1** are exposed to the channels within the single crystals, which ought to facilitate the approach by gas molecules to the reaction centers. Assuming that the sheets themselves, as covalently bonded entities, remain untouched during that process, we anticipated the product, stacked 2D polymer **2** (Figure 1 d), to be better exfoliable by LPE/LCC than 2D‐P‐**1** for two factors: First, this polymer should be neutral under appropriate pH conditions. While sonication‐assisted LPE has been widely applied to a range of charge neutral layered inorganic crystals,^8^ charged layered crystals such as clays^9^ or layered double hydroxides^10^ are commonly believed to be not exfoliable in this way due to strong electrostatic interactions. However, we note that a recent publication on a carbonate layered double hydroxide seems to suggest otherwise.^11^ Second, this polymer should be less ordered (Figure 1 d) given the severe structural changes concerning sheet packing associated with the intended post‐polymerization modification. This decrease in order should reduce interlayer forces and facilitate exfoliation.

We here report on an investigation concerning exfoliation of 2D‐P‐**1** and 2D‐P‐**2** aiming to compare these 2D polymers in terms of overall exfoliation efficiency, sheet thickness distribution, monolayer mass fraction and monolayer size in the fractions obtained from exfoliation and size selection. We will first concentrate on single crystals of 2D‐P‐**1**. They were not only subjected to LPE/LCC‐based exfoliation/fractionation, but also to a micromechanical procedure. Exfoliated products from these two methods together with already reported products from conventional wet‐chemical exfoliation were then investigated by selected area electron diffraction (SAED). This resulted in insights regarding defect formation in sheets during exfoliation and an eventual impact of the exfoliation conditions on sheet packing, which will be delineated. Thereafter we will describe how the exposure to ammonia gas under ambient conditions efficiently converts the single crystals of 2D polymer **1** into the targeted neutral analogue 2D‐P‐**2**. We will discuss to which degree powder X‐ray diffractometry (PXRD) confirms the expected decrease in long‐range order associated with this rather demanding post‐polymerization modification. Finally, we will turn to the finding that applying LPE/LCC to the new 2D polymer **2** yields quantifiable fractions with monolayers of an approximate lateral size of 130 nm as the main components (28 %). We consider this result a breakthrough for the field of exfoliation of 2D materials, and it will be explained in terms of the structural factors mentioned.

## Results and Discussion

### Liquid Exfoliation of the Pyrylium‐Based 2D Polymer 1

A number of techniques can be used to isolate mono‐layered or few‐layered sheet stacks (the latter we refer to as nanosheets) from layered bulk crystals. For example, crystals can be cleaved using adhesive tape in micromechanical exfoliation.^12^ While this procedure yields high quality samples, it suffers from very limited throughput. Large quantities of nanosheets can be obtained by exfoliation in the liquid phase.^6^ This can either be achieved based on intercalation (chemical exfoliation)^13^ or so‐called liquid phase exfoliation which relies on mechanical stress for example by shear forces or sonication.^6^ For charged layered crystals such as clays or layered double hydroxides, intercalation often accompanied with ion exchange is well established.^9^, ^10^ In this process, appropriately chosen solvents (or counterions) diffuse into the crystal resulting in an increase of the interlayer spacing which leads to swelling and subsequent delamination.9c Even though efficient exfoliation can in principle be achieved, optimization is tedious and material specific.9b, 9c This is confirmed by our previous report on 2D polymer **1**,^2^ where this delamination strategy was successfully applied, but limited to rare events. In contrast, LPE is widely applicable to (uncharged) layered crystals with little optimization required. In this process, layered crystals are immersed into a suitable solvent or surfactant solution and subjected to high energy treatment, for example by sonication. The energy input is supposed to overcome the interaction between individual layers, while the liquid environment prevents restacking when appropriately chosen. Solvent stabilization can be achieved by matching solubility parameters.^14^ Alternatively, amphiphilic additives in water can be used as surfactants.^15^ Lateral size and thickness distributions are initially broad, but can be narrowed by centrifugation‐based size selection^16^ for example by liquid cascade centrifugation.^5^ This procedure has been widely applied to a broad range of inorganic materials including *h*‐BN,^17^ transition metal dichalcogenides,14b, 15b transition metal oxides,^18^ black phosphorous,^19^ layered III–VI semiconductors (e.g. GaS,^20^ InSe^21^), IV–VI semiconductors (e.g. SnS,^22^ GeTe^23^) among others.

In the following, we compare the outcome of micromechanical exfoliation, as well as chemical exfoliation and LPE using the charged 2D‐polymer **1** primarily to assess whether the relatively simple and high yield LPE is a suitable strategy to obtain high quality nanosheets. As the mechanical forces during exfoliation may alter the chemical structure, we also place emphasis on the structural integrity of differently thick nanosheets from these three sources investigated by high‐resolution transmission electron microscopy (HRTEM) and selected area electron diffraction (SAED).

**Figure 2:**
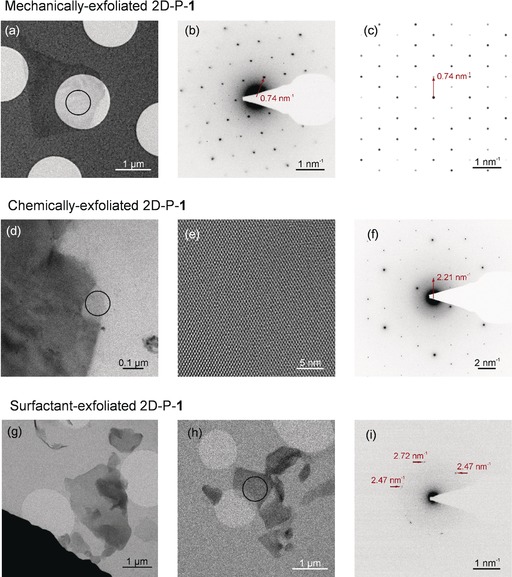
Structural characterization of single sheets of the pyridine‐based polymer **1**. a) Bright‐field TEM image of a micromechanically‐exfoliated nanosheet of the pyrylium‐based 2D polymer **1**. b) Selected area electron diffraction (SAED) of the circled area in (a) (electron dose: 3 e^−^ Å^−2^) showing good crystallinity and nearest reflections at 0.74 nm^−1^. c) Simulated diffraction pattern based on X‐ray diffraction on 2D‐P‐**1** single crystals, d) Bright‐field TEM image of a nanosheet produced from exfoliation in γ‐butyrolactone. e) HRTEM of circled area in (d) (electron dose: 800 e^−^ Å^−2^). f) SAED of circled area in (c) (electron dose: 6 e^−^ Å^−2^). Nearest reflections are observed at 2.21 nm^−1^. g,h) Bright‐field TEM images of nanosheets produced from exfoliation in aqueous surfactant. h) SAED of circled area in i) (electron dose: 0.2 e^−^ Å^−2^). A loss in crystallinity is observed after exfoliation in aqueous surfactant solution.

Figure 2 a shows a bright‐field TEM image of the pyrylium‐based 2D‐P‐**1** after micromechanical exfoliation. The corresponding SAED pattern (Figure 2 b) reveals a hexagonal symmetry with nearest reflections at 0.74 nm^−1^, which is in agreement with the simulated SAED pattern based on single crystal XRD data (Figure 1 c). Interestingly, for a nanosheet obtained after chemical exfoliation achieved by stirring in γ‐butyrolactone,^2^ the crystallinity and hexagonal symmetry are maintained (Figure 2 d–f), yet the nearest reflections are observed at 2.21 nm^−1^. We suspect that the first and second order reflections are extinguished in the solvent‐exfoliated nanosheets. Solvent intercalation can cause relaxation of stresses eventually built‐up in crystals upon single crystal to single crystal polymerizations.^24^ Nonetheless, these results demonstrate the possibility of isolating crystalline nanosheets from 2D‐P‐**1** and the sheets survive exfoliation as coherent entity. However, due to the low yield of chemical exfoliation, a statistical analysis on the representative species in the sample bears further scrutiny. We note that we do not exclude that the yield of the chemical exfoliation can be improved by further optimization. However, rather than working on such a material specific question, we aim to establish a procedure that could be efficient for a range of organic sheet stacks.

Since sonication‐assisted LPE has been shown to result in colloidally stable dispersions for a range of materials (see above) in reasonable yields without material specific optimization, we subjected 2D‐P‐**1** to this process with the aim to improve the exfoliation quality and quantity. We chose sodium cholate (SC) in aqueous solutions as surfactant stabilizer rather than organic solvents due to the ease of handling. In particular, it has been demonstrated in the case of graphite/graphene that larger populations of monolayers can be obtained in aqueous surfactant compared to solvents.^25^ The as‐sonicated dispersion was then subjected to size selection by liquid cascade centrifugation (see Supporting Information).^5^ In this iterative centrifugation process, a sample is centrifuged with subsequently increasing relative centrifugal force (RCF, expressed in units of the Earth's gravitational field, *g*). After each step, supernatant and sediment are separated and the sediments are collected for analysis, while the supernatant is centrifuged at higher centrifugal acceleration. The sediments collected at low centrifugal acceleration contain large/thick nanosheets, while the fractions collected at higher centrifugal acceleration contain smaller nanosheets. Note that the term nanosheet refers to stacks of individual 2D layers of varying thickness. Samples are labelled by indicating the lower and upper centrifugation rates. While nanosheet thickness control by LCC is poorer than by density gradient ultracentrifugation (DGU),^16^ it has the advantage of higher yield and the possibility to produce a range of size/thickness distributions which is essential to quantify the efficiency of the exfoliation process.^25^

The size‐selected fractions of 2D‐P‐**1** exfoliated in aqueous SC were first deposited on Si/SiO_2_ wafers and subjected to atomic force microscopy (AFM) to assess the result of exfoliation and size selection qualitatively. As shown by AFM imaging (Figure S1, Supporting Information), nanosheets with mostly sharp edges are obtained. As expected, nanosheets isolated at lower centrifugal acceleration are larger and thicker than nanosheets isolated at higher centrifugal accelerations, that is, the stacks contain a different number of individual layers. In fractions isolated at high centrifugal accelerations (>30k *g*), deposits with not well‐defined shape are observed along with characteristic nanosheets. While these might be due to residual surfactant, it cannot be excluded that some disintegration of the sheets occurred. The exfoliation yield of the nanosheets in the fractions was determined by a combination of gravimetry and spectroscopy (Figure S2) and showed that only 8 % of the bulk material was isolated. This is significantly lower than reported yields for graphite/graphene^25^ and WS_2_ 5 which are in the range of 20‐30% and might be an indication that LPE of 2D‐P‐**1** does not work in a way comparable to other materials that have previously been exfoliated.

To gain further insights, a sample (fraction 0.4–1k *g*) was investigated using TEM. Figure 2 g,h shows bright‐field TEM images displaying similar objects as observed with AFM. While the presence of characteristic thin sheet stacks is encouraging suggesting successful exfoliation, the SAED pattern (Figure 2 i) reveals a substantial loss of crystallinity in the nanosheets. With an electron dose of merely 0.2 e^−^ Å^−2^ (to prevent electron irradiation damage), only a few reflections could be occasionally observed, and the diffraction pattern is inconsistent with the expectation (Figure 2 d). In extreme cases, the nanosheets are completely amorphous (Figure S3). This is in contrast to delamination/exfoliation in organic solvents described above. We suspected this to be caused by an interplay of the negatively charged surfactant with the positively charged pyrylium groups of 2D‐P‐**1**, resulting in severe structural distortion within the initially crystalline matter. If this was the case, a conversion of the functional groups in the pores to neutral entities would be required for efficient exfoliation. This is addressed in the next sections.

### Post‐Polymerization Modification of 2D‐P‐1

**Figure 3:**
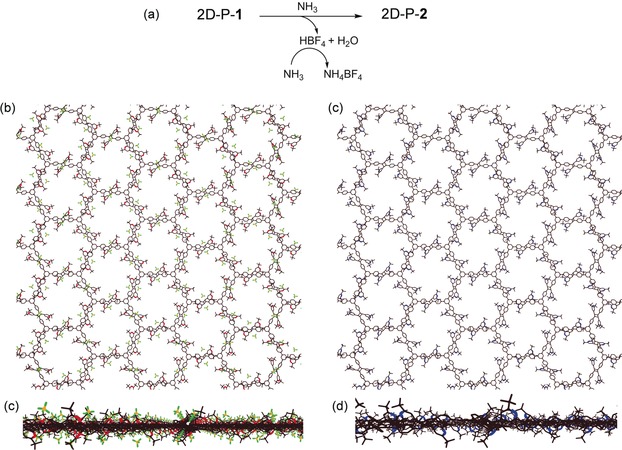
Chemical conversion of charged 2D polymer **1** into its non‐charged counterpart **2**. a) Reaction of the individual layers in a single crystal of **1** with gaseous ammonia whereby charged pyrylium (red) tetrafluoroborate groups (green‐yellow) are converted into non‐charged pyridines (blue). b,c and d,e) Same transformation as in (a) now shown as single crystal X‐ray structure in two orthogonal views for 2D polyelectrolyte **1** (b,d) and as structure model for 2D polymer **2** (c,e).

**Figure 4:**
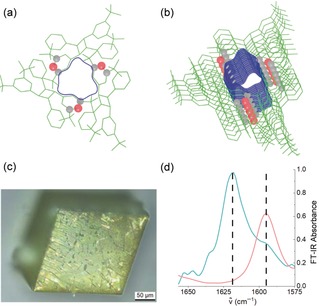
Aspects of the chemical modification. a,b) Two views of the through‐pores formed in the ABC stacked single crystals of 2D polymer **1** prior to their exposure to ammonia gas. Color code: red: pyrylium oxygen; grey: α‐C‐atoms of pyrylium. c) Optical micrograph of the reaction product. While the single crystals retain their shape, geometrical crystalline order is absent (by PXRD). d) IR spectra of 2D‐P‐**1** (cyan) and 2D‐P‐**2** (orange) showing the complete disappearance of the single band at 1620 cm^−1^ characteristic for pyrylium ions.

The post‐polymerization modification of a single crystal in a quantitative way is a challenging task. To achieve this, we build on established chemistry that suggests that pyrylium units can be converted to pyridine moieties using ammonia in near quantitative fashion.^7^ The conversion of 2D polymer **1** into its derivative 2D‐P‐**2** (Figure 3) was carried out under conditions that had been optimized in a series of model reactions (Figures S4–S10). Single crystals of 2D‐P‐**1** with sizes in the range of 50–100 μm were exposed to gaseous ammonia in a sealed glass vial at room temperature. The ABC stacked sheets of 2D‐P‐**1** in the single crystal exhibit through‐pores to the lumen of which all pyrylium ion groups are directed with one of their two α‐C‐atoms (Figures 4 a,b). At this C‐atom, the transformation of pyrylium ions to pyridine starts by the attack of the ammonia nitrogen. Thus, we expected not only a fast transport of ammonia gas into the crystals but also a facile attack at pyrylium. In fact, within seconds, the initially yellow, transparent crystals turned slightly greenish and lost their transparency (Figure 4 c). Continued exposure overnight ensured complete conversion. The reaction was analyzed by solid state ^13^C‐nuclear magnetic resonance spectroscopy. The spectra showed, in perfect agreement with model studies (see Supporting Information), the complete disappearance of the pyrylium signals at *δ*=186, 184 and 179 ppm, and the simultaneous appearance of only one broad signal in the aromatic region centred at *δ*=167 ppm (Figure S11). This one signal is as expected for the pyridine formed. Chemical shifts of pyridines all appear in a narrow shift range which our experiment cannot resolve. Because of the limited signal‐to‐noise ratio of the spectra, the conversion could not be determined with high precision, but was estimated to be >90 %. In infrared spectroscopy, measured in attenuated total reflectance (ATR), parent pyrylium tetrafluoroborate and parent pyridine show characteristic signals at 1620 cm^−1^ and 1595 cm^−1^, respectively.^26^ As these signals are virtually unchanged in the above model studies (see Supporting Information), they were taken as an indication for the proposed chemical reaction and as a measure for conversion. Figure 4 d shows the corresponding spectral regions (full spectra: Figure S12). The cyan signal at 1620 cm^−1^ of 2D polymer **1** vanishes completely to the advantage of the signal at 1695 cm^−1^ due to the target 2D polymer **2**. As the orange spectrum does not even show a shoulder at 1620 cm^−1^ the conversion seems to be (close to) quantitative.

The observed loss of transparency associated with the chemical transformation indicated loss of long‐range geometrical order. Powder X‐ray diffractometry (Figure S13) confirmed a progressive broadening of the Bragg reflections with increasing scattering angle, reminiscent of strong paracrystalline distortions.^27^ This does not come by surprise considering the severity of the action within the crystals. Qualitatively, the same phenomenon is observed when covalent organic frameworks (COFs) are internally modified.^28^ After removal of inorganic products from the powdery product by washing with ethanol/water (1:1 v/v), the geometrical order decreased even further. We assume that local disturbances due to the chemical reactions causes the 2D polymer sheets to suffer shear and to possibly wrinkle or fold. This way, the topological long‐range order, despite still present, is not reflected in geometrical order anymore and thus not “read” by the X‐ray beam.

### Liquid Phase Exfoliation of the Pyridine‐Based Polymer 2

The uncharged nature of the stacked polymer sheets of 2D‐P‐**2** suggests that established liquid exfoliation techniques can be applied to produce nanosheets.^6^ To test this, we performed LPE in aqueous surfactant solution and size selection by cascade centrifugation, as in the case of the pyrylium‐based 2D‐P‐**1**. We chose sodium cholate as surfactant stabilizer, as we expect no specific, chemical interactions to occur which allows for a direct comparison of the ease of exfoliation with other layered materials such as graphite as delineated below. In addition, we aim to establish a standard protocol that can potentially be applied to other organic sheet stacks regardless of their chemical structure. Exfoliation in solvents was also evaluated (Figure S14) and seems to be dominated by solution thermodynamics similar to graphene and other layered compounds. This is encouraging as it suggests that in contrast to 2D‐P‐**1**, 2D‐P‐**2** can be treated analogous to inorganic crystals that have been extensively studied in LPE. While solvent exfoliation seems feasible, we decided to focus on the water surfactant system in the following due to the potentially larger accessible monolayer contents,^25^ as mentioned above.

**Figure 5:**
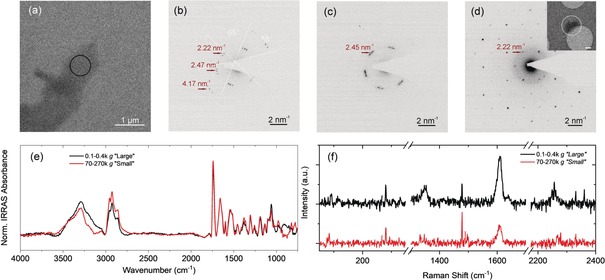
Structural characterization of liquid‐exfoliated **2** in aqueous surfactant solution. a) Bright‐field TEM image of a liquid‐exfoliated nanosheet of the pyridin‐based 2D‐P‐**2** in water surfactant. b) SAED pattern of the circled area in (a) with an electron dose of 0.1 e^−^ Å^−2^. Sharp diffraction spots are observed at 2.22 nm^−1^, 2.47 nm^−1^ and 4.17 nm^−1^. c) SAED of the same area with an accumulated dose of 0.8 e^−^ Å^−2^. Rapid degradation under the electron beam is observed. Only reflections at 2.45 nm^−1^ are observed. d) SAED pattern of the nanosheet shown in the inset (0.1 e^−^ Å^−2^, presenting hexagonal symmetry with nearest reflections at 2.22 nm^−1^. Scale bar in inset: 0.2 μm. e) Infrared‐Reflection‐Absorption spectrum (IRRAS) of two fractions of liquid‐exfoliated nanosheets enriched in larger (0.1–0.4k g) and smaller (70–270k g) nanosheets after deposition on Au‐coated Si/SiO2. f) Raman spectrum of the same samples as in e). Spectra in e,f) show no difference in vibrational modes for the different nanosheet sizes/thicknesses.

Before we assess dispersion quality and quantity in more detail, it is important to verify that the nanosheets are structurally intact. To this end, a fraction isolated at low centrifugal acceleration was subjected to TEM and SAED. Representative data is shown in Figure 5 a–d with more examples presented in the Supporting Information (Figure S15). Figure 5 a,b display a bright‐field TEM image of a nanosheet and the corresponding SAED pattern. In contrast to the surfactant‐exfoliated charged precursor polymer 2D‐P‐**1**, nanosheets of the pyridine‐based 2D‐P‐**2** exhibit higher crystallinity, which is evidenced by the sharp diffraction spots with an electron dose as low as 0.1 e^−^ Å^−2^. However, the material is extremely sensitive to electron irradiation. With an accumulated electron dose of merely 0.8 e^−^ Å^−2^, higher order reflections are vanished, signaling the loss of crystallinity. In addition, the nearest reflections shift from 2.45 nm^−1^ to 2.22 nm^−1^, which is attributed to the shrinking of the molecular network upon irradiation damage. Nonetheless, in some cases, the hexagonal structure can be clearly observed (Figure 5 d) confirming that the nanosheet crystallinity is maintained after exfoliation and size selection in surfactant. Overall, the reflections observed in SAED correspond well to the powder XRD data measured prior to exfoliation (see Supporting Information). Put differently, although structural distortions occur during ammonia treatment and post‐polymerization reaction, no further distortion is introduced during liquid exfoliation, demonstrating the non‐invasive nature of our technique. Note that, thinner nanosheets isolated at higher centrifugal acceleration could no longer be characterized by SAED, presumably due to their extreme sensitivity to electron irradiation.

To confirm that smaller/thinner nanosheets isolated at higher centrifugal acceleration are structurally intact, the dispersions were deposited on Au‐coated Si/SiO_2_ wafers (Figure S16) and subjected to vibrational spectroscopy. The deposition as homogeneous films enabled characterization by infrared‐reflection‐absorption spectroscopy (IRRAS). In IRRAS, only bands with a dipole moment perpendicular to the surface are excited. As such, a direct comparison to the ATR‐FTIR of the powder crystals is not possible. However, different size‐selected dispersions can be compared. Dispersions containing larger/thicker nanosheets (0.4–1k g) and smaller/thinner nanosheets (70–270k g) were subjected to the measurements (see Supporting Information). No significant spot‐to‐spot variations across one sample were observed (Figure S17). The IRRAS absorbance spectra normalized to the highest intensity vibrational mode (Figure 4 e) evidence that the small and large nanosheets show the same vibrations with insignificant variations in relative intensities. This suggests that no structural damage was introduced in the fraction of small/thin nanosheets. An assignment of the peaks is beyond the scope of the publication.

A similar picture is obtained by Raman spectroscopy (532 nm excitation), with both samples showing the same vibrational modes across the entire spectral region (Figure 5 f). The measurement again confirms that potential structural damage is below the detection limit. Due to the non‐resonant excitation, the signal is very weak and long accumulation times are required. In addition, care must be taken not to damage the material by the localized laser excitation. Also, note that the Raman measurements were only possible after deposition of the nanosheets on the Au‐coated substrate. Otherwise (e.g. on the bulk crystal), heating affects and laser‐induced degradation dominate the spectral response masking vibrational modes.

**Figure 6:**
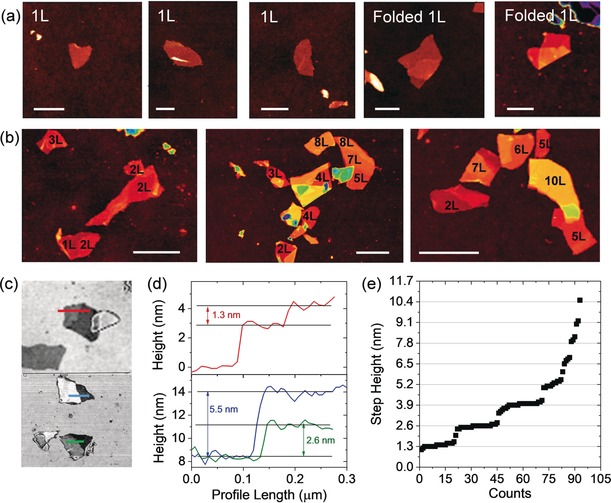
Atomic force microscopy on liquid‐exfoliated nanosheets of 2D‐P‐**2**. a,b) Representative AFM images of 1 L nanosheets (a, scale bar 200 nm) and few‐layered nanosheets (b, scale bar 500 nm). Few‐layered nanosheets often have characteristic terraces c) AFM image of nanosheets with terraces in black/white contrast for better visualization. d) Height profiles along the lines in (c). e) Height of steps associated with terraces (not from nanosheet to substrate) plotted in ascending order. The steps are always a multiple of 1.3 nm, which is assigned to the apparent height of one layer.

The nanosheet morphology was further examined by atomic force microscopy (AFM, Figure 6 and S18). We find nanosheets with shapes characteristic for LPE. The thinnest (thickness ≈2.5 nm, Figure 6 a) have a homogeneous surface, usually rather soft edges and are often folded. In contrast, slightly thicker nanosheets (Figure 6 b) have sharper edges, are less folded and have characteristic terraces. These are best visualized in black and white contrast (Figure 6 c) with profiles along the sheets displayed in Figure 6 d. In analogy to previous work on LPE, we can use a statistical analysis of the height of these steps to convert the apparent AFM thickness to a layer number.19a, 29 This is required, as the apparent AFM height of LPE nanosheets is overestimated compared to the theoretical thickness due to adsorbed/intercalated solvent/surfactant as well as contributions from capillary forces and material dependent adhesion, in particular for measurements under ambient conditions.^30^ The height of the steps (only measured from nanosheet to nanosheet and not nanosheet to substrate) are plotted in ascending order in Figure 6 e and show that they are a multiple of 1.3 nm. We conclude that this is the apparent height of one layer. Note that this value is in between 0.95 nm obtained for graphene29b, 31 1.9 nm found for MoS_2_ 29a in accordance with the structures of the materials.

In principle, we can use the 1.3 nm step height to determine the layer number N of each sheet. However, as previously proven for MoS_2_ 29a and graphene,29b the monolayers appear ≈1 nm thicker than the step height of one layer, mostly due to solvent/surfactant trapped between nanosheet and substrate. The observed 2.5 nm of the thinnest 2D polymer nanosheets is consistent with this and is thus assigned to monolayers. Additional 1.3 nm are added per additional layer according to the step height.

With this knowledge, the exfoliation process can be investigated in more detail. For example, we can analyze the folding statistically as function of layer number (Figure S19). We find that ≈25 % of the monolayers are folded with some folding still being observed for 2–4 layers, but not when the layer number exceeds 5 layers. This is a clear manifestation of the thickness dependent stiffness of the polymer.

### Quantification of the Liquid Phase Exfoliation of 2

In the following, we describe the characteristics of 2D polymer dispersions that can be isolated from an as‐sonicated stock by liquid cascade centrifugation. The procedure is discussed in more detail in the Supporting Information (Figure S20–21). A similar initial cascade as for polymer 2D‐P‐**1** was used. In addition to the samples produced by this standard cascade, a subset of the dispersions was centrifuged overnight in a secondary cascade to achieve further monolayer enrichment.

**Figure 7:**
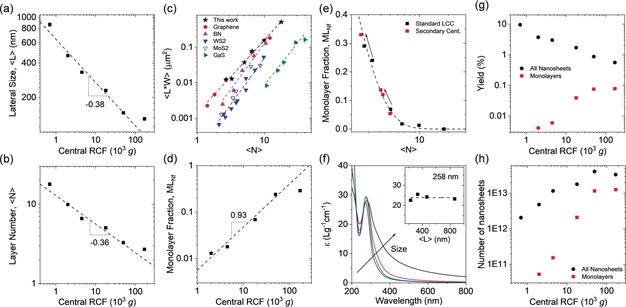
Characterization of fractions obtained after liquid exfoliation and centrifugation‐based size‐selection. a,b) Plot of mean lateral size, ⟨L⟩ (a) and mean layer number ⟨N⟩ (b) determined by AFM statistics as function of the midpoint of centrifugal acceleration (RCF) used in cascade centrifugation. c) Plot of mean nanosheet area (expressed as length L, multiplied by width W) as function of mean layer number for a range of materials. The graphene reference was established in the course of this work, while data for BN, MoS_2_, WS_2_, BN and GaS was extracted from literature.^5^, 17b, 20, 33 d) Plot of monolayer number fraction as function of central RCF. e) Plot of monolayer number fraction as function of mean layer number. Data from the standard cascade (black) and secondary cascades (red) are included demonstrating the monolayer enrichment by secondary cascades. f) Extinction coefficient as function of wavelength for different fractions. Nanosheet concentration was determined gravimetrically. g) Plot of extinction coefficient, *ϵ*, at different wavelengths as function of nanosheet size. The size‐invariant coefficient at 258 nm was chosen for the assessment of the yield. h) Plot of nanosheet yield of all nanosheets and the monolayer content as function of central RCF.

To gain insights into the size/thickness distribution of the nanosheets, each fraction was subjected to statistical AFM. In each case, the longest dimension (L), the dimension perpendicular to L (denoted as width, W) and the thickness were measured for 200–250 nanosheets. The thickness was converted to the layer number as discussed above, the lateral dimensions were corrected for cantilever broadening and pixelation by a previous calibration.^32^ Representative images of each fraction and distribution histograms are shown in the Supporting Information (Figure S22–S25). From this analysis, mean lateral size ⟨L⟩, layer number ⟨N⟩, and monolayer content described as monolayer number fraction, ML_NF_ and monolayer volume fraction, ML_Vf_, respectively, were obtained as summarized in Table 1. The data is graphically presented in Figure 7 (and Figures S26,27). Figure 6 b and 7 a plot the mean length ⟨L⟩ and mean layer number ⟨N⟩ as function of the midpoint of RCF of the cascade. In both cases, a well‐defined power law scaling is observed which is, as expected, similar to other (inorganic) 2D materials.^5^, 17b, 20 This is useful for practical reasons, as it allows one to set the centrifugation boundaries accordingly when a specific size/thickness is targeted.

**Table 1:** Mean nanosheet dimensions, monolayer contents and yields in the fractions produced by liquid cascade centrifugation. The table shows data from both the standard cascade as well as secondary LCC. The arithmetic mean of the layer number ⟨N⟩, lateral size ⟨L⟩ and monolayer content (expressed as both number fraction ML_NF_ and volume fraction ML_Vf_), yield, monolayer yield and number of nanosheets is summarized.

	Primary 0.4–1k *g*	Primary 1–3k *g*	Primary 3–6k *g*	Primary 6–30k *g*	Primary 30–70k *g*	Primary 70–270k *g*
⟨N⟩	18	10	6.6	5	3.3	2.7
⟨L⟩ [nm]	870	465	330	230	145	130
ML_NF_	0	0.013	0.018	0.069	0.24	0.29
ML_Vf_	0	0.001	0.002	0.023	0.087	0.14
Yield [%]	9.5	3.7	3	1.7	0.87	0.56
ML Yield [%]	0	0.004	0.006	0.039	0.076	0.078
# of all nanosheets	2.1×10^12^	4.8×10^12^	1.2×10^13^	1.8×10^13^	4.1×10^13^	3.4×10^13^
# of ML	0	5.2×10^10^	1.5×10^11^	2.1×10^12^	1.2×10^13^	1.3×10^13^

	Secondary 6–30k 1k14hS		Secondary 6–30k 1k14hSed	Secondary 6–30k 1k14hS 7k1hS	Secondary 30–70k 6k14hS	Secondary 30–70k 6k14hSed
⟨N⟩	4.2		6.6	4	2.5	5
⟨L⟩ [nm]	212		237	218	138	177
ML_NF_	0.12		0–0.01	0.14	0.33	0.054
ML_Vf_	0.046		∼0.001	0.057	0.18	0.016
Yield [%]	0.87		0.77	0.56	0.17	0.42
ML Yield [%]	0.040		0.0008	0.032	0.030	0.007
# of all nanosheets	1.5×10^13^		4.3×10^12^	9.5×10^12^	1.1×10^13^	8.9×10^12^
# of ML	2.8×10^12^		2.8×10^10^	2.2×10^12^	5.0×10^12^	7.1×10^11^

To compare the exfoliation quality to other materials, the nanosheet area (expressed as ⟨L W⟩) is plotted as function of layer number ⟨N⟩ in Figure 7 c. Data for WS_2_, MoS_2_, BN and GaS is extracted from the literature,17b, 20, 29a, 33 while the graphene data was collected from a reference experiment under similar experimental conditions to ensure maximum comparability (Figure S28, Table S3). In all cases, a well‐defined scaling of lateral size with layer number is observed. Importantly, the curves are horizontally offset from each other depending on the material. This indicates that some materials are easier to exfoliate than others, reflected in larger areas for the same nanosheet thickness. A recently developed model suggests that this is a result of nanosheet exfoliation and tearing both occurring with equal energy inputs.^25^ As such, the lateral size–thickness aspect ratio reflects the ratio of edge to surface energy associated with scission and exfoliation, respectively. Importantly, the data from the 2D polymer collapses on the same curve with graphene (albeit with a vertical offset due to a different density of the materials influencing the centrifugation). This evidences that 2D polymer **2** is as “exfoliatable” as graphene. This is interesting because the intralayer bonding strength in the 2D polymer **2** is likely weaker than that of the conjugated sp^2^ carbon framework of graphene. However, at the same time, the surface of the individual sheets of 2D‐P‐**2** is less smooth than graphene (see Figure 3 e) which reduces the interlayer binding energy. This can result in a similar edge to surface energy ratio as found in graphene as corroborated by the experimental data. This is an important finding, as it suggests that, in contrast to inorganic layered crystals, rational synthesis can possibly be used to design organic sheets stacks with minimized interlayer bonding in the future that will be easier to exfoliate.

The monolayer content is analyzed in more detail below. A plot of ML_NF_ as function of central RCF is shown in Figure 7 d. As with ⟨L⟩ and ⟨N⟩, we find a power law scaling with monolayer number fractions of close to 0.3 accessible in the last step (after ultracentrifugation at 270 000 *g*). This corresponds to a monolayer volume fraction of 0.14 (Figure S26 e). In samples produced from standard cascade centrifugation, lateral dimensions and layer number are linked as implied by Figure 7 a,b. We note that this is mostly because laterally larger nanosheets require more energy to overcome the interlayer interactions so that this behavior is also observed when analyzing individual nanosheets (Figure S26b). This has two important implications: i) nanosheets in the monolayer‐rich fraction are laterally also relatively small (130 nm); ii) it is extremely challenging to produce nanosheet dispersions with the same mean thickness, but different lateral size and vice versa. To overcome this limitation, secondary cascades involving overnight centrifugation at lower centrifugal acceleration than the initial lower centrifugation boundary were previously suggested as potential strategy.^5^ Applying this principle to fractions of the 2D polymer, we were able to decouple the length–thickness relationship (Figure S27). Importantly, this resulted in further monolayer enrichment with minimal sacrifice of the lateral nanosheet dimensions. This enrichment is illustrated by the plot of ML_NF_ as function of ⟨N⟩ in Figure 7 e. In general, we find an exponential scaling of monolayer content with layer number as previously documented for WS_2_.^32^ In the plot, data from the secondary cascade is shown in red, the blue arrow indicates the starting point. In this way, a monolayer number fraction of 0.33 was achieved with slightly larger nanosheets (138 nm) compared to the standard cascade. Importantly, the procedure did not require an ultracentrifuge and is thus experimentally more accessible.

In the following, we address the question of the yield of the exfoliation process. While nanosheet concentrations in high mass fractions are accessible gravimetrically after filtration and weighing, the reliability is limited in the case of the monolayer‐rich, but lower mass fractions. To determine nanosheet concentrations reliably, it is thus required to know the extinction coefficient *ϵ*, which is known to be dependent on nanosheet size for other layered materials due to edge and confinement effects as well as scattering.^5^, 17b, 19a, 20, 29a Extinction coefficient spectra for different fractions of 2D‐P‐**2** are plotted in Figure 7 f. As expected, systematic changes with nanosheet size are observed as discussed in more detail in the Supporting Information (Figure S29–31). In brief, extinction spectra of colloidal nanomaterials are a combination of absorbance and light scattering. Contributions from light scattering can be eliminated by measurements in an integrating sphere which yield true absorbance spectra. From this data (Figure S29), it is clear that the 2D‐P‐**2** is non‐resonant at >400 nm so that any intensity in extinction spectra is due to light scattering. This allows us to classify nanosheets of 2D‐P‐**2** as a wide band gap semiconductor with an optical gap of ≈3.55 eV. As analyzed and modelled in depth recently,^34^ the wavelength‐dependent scattering of randomly‐oriented platelets in the non‐resonant regime is characterized by a power law (as observed here) with exponent and pre‐factors being dependent on nanosheet volume and longest dimension, respectively. The data on 2D‐P‐**2** is consistent with these observations.

We note that not only extinction spectra show size‐dependent changes due to scattering, but also the absorbance spectra change. This demonstrates that similar edge and confinement effects previously observed for inorganic 2D materials^5^, 17b, 19a, 20, 29a are at place. For example, peak intensity ratios in absorbance change due to edge effects that result in absorbance coefficients that are different at edge and center regions. Furthermore, confinement and dielectric screening lead to systematic shifts of excitonic peak positions. Both effects are observed for 2D‐P‐**2** (Figure S29,30). This shows that the optical properties of exfoliated 2D‐P‐**2** exhibit a similar thickness dependence known for inorganic 2D materials, that is, the properties are governed by layer number. An understanding of the optical spectra is of great practical use, as it allows us to establish quantitative size and thickness metrics based on optical spectra for rapid size assessment. Examples are shown in Figure S31. Peak intensity ratios in the resonant regime scale with the lateral size due to edge effects, while ratios in the non‐resonant to resonant regime of the extinction spectra reflect the scattering strength of the nanosheets which depends on the longest dimension.

The understanding of the optical spectra can be used to find a wavelength, where the extinction coefficient is size‐invariant. This is the case at 258 nm, where the associated extinction coefficient of 24 L g^−1^ cm^−1^ can be used for an assessment of the nanosheet concentration independent of the nanosheet size (Figure 7 f, inset). With this knowledge, we determined the yield of both exfoliation and size selection. This is plotted as function of RCF in Figure 7 g for both all nanosheets and the monolayers in each fraction (Table 1). The yield of all nanosheets decreases roughly as power law with RCF and hence size. As a consequence of the increased monolayer population with increasing RCF, the ML yield increases steadily. The overall yield of exfoliated nanosheets over all fractions and sizes is 20 %. This is identical to the graphene yield in our reference experiment (Figure S28) and significantly higher than in the case of the pyrylium‐based polymer **1**. The yield of the ML‐rich fraction is ≈0.6 % corresponding to ≈0.6 mg. The total monolayer yield is in the order of 0.2 %. While the yields appear low at first glance, it should be noted that the previously unexfoliated material can be recycled in a second exfoliation yielding a very similar result as in the initial exfoliation (Figure S32–33). We thus anticipate that the achievable mass can be increased further by optimizing the exfoliation or increasing the initial concentration of the starting material14a which was rather low for an LPE process (4 g L^−1^) in this case. With knowledge of the structure, nanosheet dimensions and mass, it is possible to calculate the number of nanosheets in each fraction (see Supporting Information). In contrast to the total yield, the total number of sheets increases with increasing RCF (Figure 7 h). Importantly, the plot demonstrates that indeed large quantities of nanosheets in the range of tens of trillions of sheets are produced in each fraction. In the monolayer‐rich fractions isolated at high RCF, >10^13^ monolayer nanosheets are obtained.

It should be noted that liquid exfoliation in combination with size selection also leads to a purification of the starting material. While characterization of the powder suggested a near quantitative post polymerization modification, we find evidence of some residual pyrylium‐based polymer **1** in the fraction of the material that was removed as unexfoliated crystallites in the first centrifugation step (Figure S34,35). As discussed above and in the Supporting Information, the charged pyrylium‐based 2D‐polymer **1** cannot be exfoliated efficiently in the anionic surfactant sodium cholate. For example, only 8 % of the starting material of the 2D‐polymer **1** are exfoliated opposed to 20 % in the case of the modified pyridine‐based polymer **2**. Thus, it is likely that traces of the incompletely modified 2D‐P‐**1** are enriched in the sediment at low centrifugal accelerations and thus separated from well‐exfoliated sheet stacks of **2** that remain in the supernatant. From optical absorbance (Figure S35), we estimate that ≈3 % of 2D‐P‐**1** was not completely converted to 2D‐P‐**2**.

## Conclusion

In summary, post‐polymerization chemical transformation of the amply available 2D‐P‐**1** allows to create a novel 2D polymer **2** from an existing one. This conversion reduces the initially long‐range ordered packing, but leaves individual 2D polymer sheets intact. Furthermore, it proceeds in virtually quantitative yield. The synthesis just requires exposure of single crystalline starting material to gaseous ammonia. It can readily be performed in gram quantities with further potential for effortless scale up. Making the ordered covalent network structure of a 2D polymer from scratch is demanding in terms of both design and proper execution. To now have a way to convert one 2D polymer into another is therefore an important accomplishment from the vantage point of polymer synthesis. 2D‐P‐**2** contains pores, as its precursor, which are monodisperse and evenly spaced over the entire sheet plane. They make up most of the surface area. Since the sheets are only approximately 1 nm thick, mechanically strong and expose pyridine nitrogen atoms that can be reversibly protonated to the desired degree by the applied pH, the 2D polymer 2D‐P‐**2** comprise important features of an ideal membrane for gas and, particularly, ion separation applications.

The exfoliation of the single crystalline 2D polymers 2D‐P‐**1** and 2D‐P‐**2** was investigated concerning nanosheet integrity and efficiency. Although sheet integrity did not turn out to be of a major concern in both cases, only 2D‐P‐**2** led to satisfactory results regarding exfoliation efficiency, monolayer content and sheet size. Only the uncharged 2D‐P‐**2** could be exfoliated in aqueous surfactant solution, where higher degrees of exfoliation^25^ are expected. In case of the positively charged 2D‐P‐**1,** a loss of crystallinity is observed when using this efficient exfoliation medium. AFM statistical analysis of size‐selected fractions of 2D‐P‐**2** obtained from sonication‐assisted LPE in combination with monolayer enrichment by cascade centrifugation showed that exfoliation quantity (i.e., nanosheet yield) and quality (i.e., relationship between lateral size and layer number) are comparable to graphene and hence superior to layered inorganic materials. A fraction of 2D‐P‐**2** amounting to 2.2 mg of total sheet mass was readily obtained, which contained 29 % monolayers (by number) with an average length of 130 nm. This corresponds to 1.3×10^13^ monolayer sheets in this fraction. This finding is unique in terms of both methodological development to precisely assess exfoliation quantity and quality, as well as the level of enrichment of monolayers of that size.

Importantly, this is the first demonstration that LPE and LCC can be used to produce monolayer 2D polymer sheets in dispersion in large quantities. This implies that the knowledge gained from other materials (e.g. graphite, MoS_2_ etc.) over the past few years can be applied in a straightforward manner. This includes scale up of the exfoliation for example by shear exfoliation,29b ball milling^35^ or microfluidization^36^ to increase the accessible quantities of the exfoliated sheets and enable further solution processing and printing.^37^ Possibly, a combination of different exfoliation strategies based on both chemical (intercalation) and mechanical exfoliation mediated through various sources on energy input can result in a larger population of large and thin sheets in the sample (even though this has currently not been demonstrated for other layered crystals). In addition, other size selection techniques^16^ such as density gradient ultracentrifugation, which has the potential to achieve sorting strictly by layer number, can potentially be applied. The low density of the porous organic stacks should actually facilitate isopycnic separation in a density gradient. Furthermore, one can envisage to design surfactants with a specific chemical interaction. In the case of graphene, surfactants with an extended π‐system (such as pyrenes) were shown to improve exfoliation quality and quantity.^38^ In the case of 2D‐P‐**2**, one could think of amphiphiles containing pyridine units due to the relatively strong interactions between pyridine rings^39^ or amphiphiles containing pyridinium and quinolinium moieties.

Furthermore, our work suggests that also other related structures such as 2D covalent organic frameworks (COFs)^40^ can be exfoliated in the same way potentially giving access to hundreds of novel materials with chemically designed properties in a solution processable form. Last but not least, the exfoliation and ability to deposit the sheet stacks on arbitrary substrates gives access to characterization techniques, for example Raman spectroscopy, that can often not be applied to the bulk crystals due to heating effects and beam damage.

## Conflict of interest

The authors declare no conflict of interest.

## Supporting information

As a service to our authors and readers, this journal provides supporting information supplied by the authors. Such materials are peer reviewed and may be re‐organized for online delivery, but are not copy‐edited or typeset. Technical support issues arising from supporting information (other than missing files) should be addressed to the authors.

Supplementary
