# Supplementary material for: Enriching and Quantifying Porous Single Layer 2D Polymers by Exfoliation of Chemically Modified van der Waals Crystals
Source: Angew Chem Int Ed Engl. 2020 Jan 23;59(14):5683–95. doi: 10.1002/anie.201912705 (PMC7154524; doi:10.1002/anie.201912705)
Supplement: Supplementary file 1 — Supplementary [file ANIE-59-5683-s001.pdf]

Angewandte Chemie
Eine Zeitschrift der Gesellschaft Deutscher Chemiker

Supporting Information

Enriching and Quantifying Porous Single Layer 2D Polymers by Exfoliation of Chemically Modified van der Waals Crystals

Ralph Z. Lange[+], Kevin Synnatschke[+], Haoyuan Qi, Niklas Huber, Gregor Hofer, Baokun Liang, Christian Huck, Annemarie Pucci, Ute Kaiser, Claudia Backes, and A. Dieter Schlüter**

anie_201912705_sm_miscellaneous_information.pdf

Table of Contents

I. METHODS 2
II. LPE OF THE PYRYLIUM-BASED POLYMER 7
II.1 Atomic force microscopy 7
II.2 Extinction/absorbance spectroscopy 7
II.3 Transmission electron microscopy 10
III. POST-POLYMERIZATION MODIFICATION 11
III.1 Procedures for the synthesis of model compounds and the model reactions 11
III.1.1 Synthesis of 2,6-di-tert-butyl-4-(4-methylstyryl)pyrylium tetrafluoroborate A [6] 11
III.1.2 Synthesis of 4,4'-(2,4-di-p-tolylcyclobutane-1,3-diyl)bis(2,6-di-tert-butylpyrylium) bis(tetrafluoroborate) B 15
III.1.3 Synthesis of 4,4'-(2,4-di-p-tolylcyclobutane-1,3-diyl)bis(2,6-di-tert-butylpyridine) C 18
III.2 Details of model studies 21
III.3 Details of the post-polymerization modification of 2D polymer **1** 27
IV. EXFOLIATION OF THE PYRIDIN-BASED POLYMER 34
IV.1 Exfoliation in solvents 34
IV.2 Transmission electron microscopy 38
IV.3 IR-Reflection-Absorbance Spectroscopy (IRRAS) 39
IV.4 AFM analysis nanosheet folding 41
IV.5 Size selection procedures 43
IV.5.1 Standard cascade 43
IV.5.2 Secondary cascade 44
IV.6 Size selection characterization 45
IV.6.1 AFM 45
IV.6.2 Scaling of nanosheet dimensions 48
IV.7 Graphene reference experiment 52
IV.8 Size dependent optical properties – quantitative length and thickness metrics 54
IV.9 Sediment recycling 62
IV.10 Calculation of the number of nanosheets 64
IV.11 Characterization of the fraction of unexfoliated 2D polymer **2** 65
V. REFERENCES 68

I. METHODS

*Synthesis of 2D polymer **1***

2D-P-**1** was prepared according to a method published previously by us.[1] The multi-step synthesis is based on a photochemical single-crystal to single-crystal polymerization. The monomer is synthesized through the condensation of a trifunctional aldehyde with a 4-methyl pyrylium salt in the last step and was optimized to give up to 35g of the target monomer (denoted as monomer **3** in [1]). We note that this monomer is now commercially available with TCI chemicals (CAS 2056254-18-1). Photo-reactive single crystals were obtained after recrystallization from methanol/acetonitrile 9:1 v/v with controlled cooling (60 °C to r.t. over 24 - 72h; method 2 of the SI[1]). Photo-polymerization to obtain 2D-P-**1** from the single crystals of the monomer was carried out with green LED light (532 nm).

*Synthesis of 2D polymer **2***

A few mg of dry single crystals 2D-P-**1** were placed with a spatula in a 4 mL vial. This vial was placed inside a 25 mL vial containing a bath of 3 mL conc. aqueous ammonia, which was then sealed airtight with a screw cap. An instant change of color from yellow to light tan was observed. After 24 h, the vial was vented, whereupon the crystals turned green and residual ammonia gas was removed by applying vacuum for 1 h. To remove byproducts (*e.g.* NH_4BF_4), crystals where washed with EtOH/H_2O (2 × 5 mL 1:1 v/v) and dried under reduced pressure. Quantitative conversion was indicated by CP/MAS ^{13}C NMR spectroscopy (Figure S11) and IR spectroscopy (Figure S12).

Exfoliation

Dispersions were prepared by probe sonicating the powder of **1** and **2**, respectively (initial concentration 4 gL^{-1}) in an aqueous surfactant solution (sodium cholate, SC, Sigma Aldrich, order number C1254-100G). The polymer powder was immersed in 25 mL of aqueous surfactant solution (C_{SC} = 2 g/L) in a 50 mL plastic centrifuge tube (VWR, order number 525-0402). The tube was mounted in a water bath connected to a chiller system to maintain the external temperature at 5°C. A tapered sonication microtip was lowered to the bottom of the beaker, then raised 2 cm above the

vial bottom. The mixture was sonicated by probe sonication (Sonics VXC-500) for 5 h at 40 % amplitude with a pulse of 6 s on and 6 s off.

Size selection –standard cascade

For the initial size selection, the as-obtained dispersion was subjected to liquid cascade centrifugation with sequentially increasing rotational speeds.[2] All centrifugation runs were performed at 10°C in 1.5 mL Eppendorf vials for 2 h. For centrifugal accelerations (expressed as relative centrifugal force, *RCF* in units of the earth's gravitational field, *g*) < 6,000 *g*, a Hettich Mikro 220R centrifuge equipped with a fixed-angle rotor 1195-A was used; above 6,000 *g*, a Beckman Coulter Avanti XP centrifuge with a JA25.50 fixed angle rotor was used. For the centrifugation at 270000 g, a Beckman Coulter OPTIMA XPN-80 ultracentrifuge with a swinging bucket rotor (SW40Ti, 12.5 mL Beckman polypropylene tubes) was used. To prepare the initial dispersions containing different nanosheet sizes, unexfoliated material was first removed by centrifugation at 400 *g*. The supernatant after this step was centrifuged at 1,000 *g* and supernatant and sediment separated. The supernatant was subjected to centrifugation at higher centrifugal acceleration as detailed below and the sediment collected in overall ~1.5 mL of fresh SC (c_{SC}=0.1 gL^{-1}). The centrifugation was continued with 3k *g*, 6k *g*, 30k *g*, 70k *g*, 270k *g*. After each step, the sediments were collected in fresh SC, while the supernatant was subjected to centrifugation at the next higher centrifugal acceleration. The final supernatant was discarded. The sample nomenclature of each size-selected dispersion refers to the lower and upper boundary of the centrifugation, respectively.

Size selection –secondary cascade

The dispersion 6-30k *g* was first centrifuged for 14 h at 1k *g*. Both sediment and supernatant were collected and analyzed. The supernatant was then centrifuged again for 7 h at 7k *g*. and the supernatant collected and analyzed. In addition, the dispersion 30-70k *g* was centrifuged for 14 h at 6k *g*. Both sediment and supernatant were collected and analyzed.

Characterization

^{1}H NMR and ^{13}C NMR measurements were performed on a Bruker Avance 300 MHz spectrometer (Bruker BioSpin AG, Germany). Optical Micrographs were recorded on a Leica DM2000 microscope with Leica DMC 2900 digital camera (Leica Microsystems GmbH, Germany).

Infrared spectroscopic analyses were carried out using an attenuated total reflection (ATR) - Fourier transform infrared (FTIR) spectrometer (Bruker Optics Alpha system with a built-in diamond ATR). The samples were powdered by pushing down the diamond on the sample against the quartz detector window at room temperature. The background was recorded always right before sample measurement with the diamond released and then automatically subtracted. OPUS 6 from Bruker was used for processing. The data represent the average of 128 scans in the wavenumber range between 3500 – 400 cm^{-1} at a resolution of 4 cm^{-1}. Intensities were categorized as followed: vs = very strong (0 – 10% T), s = strong (10 – 40% T), m = medium (40 – 70% T), w = weak (70 – 90% T), vw = very weak (90 – 100% T).

MALDI-TOF-MS was performed on a Bruker Solarix spectrometer by the ETH Laboratory of Organic Chemistry mass spectrometry service.

XRD measurements and structure determinations were performed by the ETH Small Molecule Crystallography Center. Measurements were performed on a Rigaku Oxford Synergy instrument with κ-geometry. An Incoatec Microsource with copper target was used as a source and the beam monochromatized with a graphite monochromator to provide $Cu_{K\alpha1}$ radiation (λ = 1.71073°). A Dectris Pilatus 300K pixel detector recorded the diffracted intensities. Data reduction was carried out with CrysalisPro[3] structure solution and refinement with the shelx suit[4] integrated in OLEX2[5] with least-squares refinement against intensities.

Wide-angle powder X-ray diffraction patterns were recorded with a Panalytical X'Pert PRO MP diffractometer in Bragg-Brentano geometry equipped with a 1D detector. A copper X-ray tube was used as a radiation source (λ = 1.54056A°) and Ge(111) as a monochromator. The respective powder was distributed on a silicon zero diffraction plate and placed into the PXRD auto sampler. All substances were measured over a 2θ-range from 5° to 65° with a step size of 0.02° and an exposure time of 90 s per 0.1°.

TEM measurements were conducted on an image-side corrected FEI Titan 80-300 operated at 300 kV. The microscope is equipped which a CEOS hexapole aberration-corrector which corrects the geometrical axial aberrations up to the 3rd-order. Data acquisition was conducted on a Gatan UltraScan CCD camera. And data analysis was carried out using Gatan DigitalMicrograph software.

Atomic force microscopy (AFM) was carried out on a Dimension ICON3 scanning probe microscope (Bruker AXS S.A.S.) in ScanAsyst in air under ambient conditions using aluminium coated silicon cantilevers (OLTESP-R3). The concentrated dispersions were diluted with water to optical densities <0.1 across the resonant spectral region. A drop of the dilute dispersions (20 μL) was deposited on a pre-heated (180 °C) Si/SiO_2 wafers (0.5x0.5 cm^2) with an oxide layer of 300 nm. After deposition, the wafers were rinsed with ~5 mL of water and ~5 mL of isopropanol. Typical image sizes were 20x20 (for 0.4-1k *g*, 1-3k *g*, 3-6k *g*) or 8x8 μm^2 (all other dispersions) at scan rates of 0.5 Hz with 1024 lines per image.

Optical extinction was measured on a Varian Cary 6000i in quartz cuvettes with a pathlength of 0.1 cm in 0.5 nm increments. Samples were diluted to extinction values of <0.5 across the entire spectral region. To measure absorbance, the dispersions were placed in the center of an integrating sphere (external DRA-1800). Nanosheet concentrations of a subset of dispersions were determined gravimetrically after filtration through alumina membranes, washing with ~ 500 mL of water, drying and weighing.

For IRRAS and Raman measurements, the nanosheets were deposited onto thermally evaporated Au (150 nm) on silicon wafers by dip coating. Infrared reflection-absorption spectroscopy (IRRAS) was performed using a Bruker IFS 66v/s Fourier-transform (FT) IR spectrometer equipped with a liquid nitrogen cooled MCT detector. The samples were mounted to a homemade reflection unit inside the sample compartment of the spectrometer. Samples were illuminated by polarized light emitted from a thermal light source under an angle of incidence of 83° with respect to the surface normal. The whole beam path was evacuated to 5 mbar in order to avoid atmospheric water and carbon dioxide absorption. Spectra were recorded with a resolution of 4 cm^{-1} and 500 scans. Reflectance spectra were measured for p- and s-polarized light and were divided by a background spectrum of a bare gold substrate. According to the surface selection rules, measurements with s-polarized light do not yield any signal from the molecular layer and can therefore be used to determine the baseline. Therefore the normalized relative reflectance

$$R_{\mathrm{norm}} = \frac{R_{\mathrm{p}}(\mathrm{polymer})/R_{\mathrm{p}}(\mathrm{Au})}{R_{\mathrm{s}}(\mathrm{polymer})/R_{\mathrm{s}}(\mathrm{Au})},$$

where R_{p} and R_{s} denote the p- and s-polarized reflectance, is shown.

Raman spectroscopy was performed with a Renishaw InVia microscope with 532 nm excitation laser under ambient conditions. The Raman emission was collected by a 50×, long working distance objective lens in streamline mode and dispersed by a 2400 l/mm grating with 0.5 % of the laser power (<0.1 mW). Note that the laser power has to be kept as low as possible to avoid heating and sample decomposition. Typical acquisition times were 42 h (!) per spectrum.

II. LPE OF THE PYRYLIUM-BASED POLYMER

II.1 Atomic force microscopy

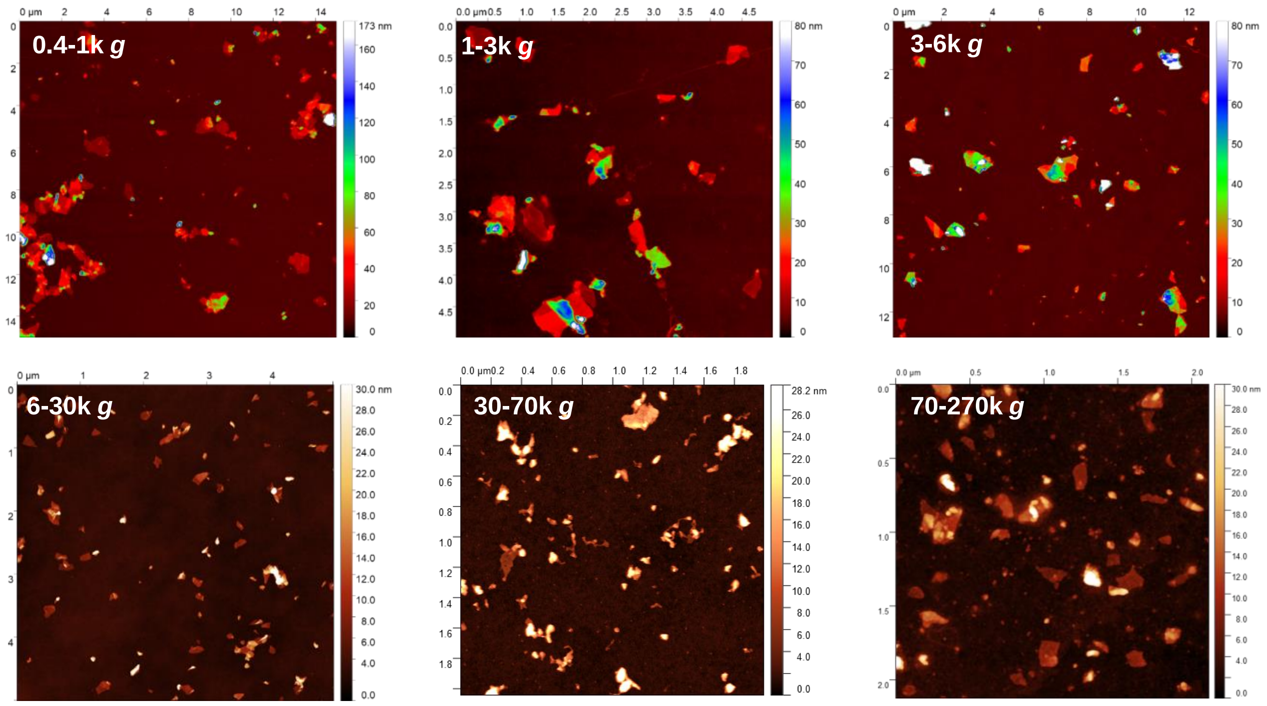

Figure S1: Atomic force micrographs of size-selected fractions of the pyrylium-based 2D polymer **1** after exfoliation in aqueous sodium cholate. In particular in the fractions isolated below 30k *g*, nanosheets with well-defined shapes and shark edges are found. Lateral size and thickness decreases with increasing centrifugal acceleration as expected. Fractions isolated at RCF>30k *g* contain a lot of deposits of ill-defined shapes. While some of these might arise from surfactant impurities, the worm-like structures in the 30-70k *g* sample hint to some degradation that occurred on exfoliation.

II.2 Extinction/absorbance spectroscopy

To determine the yield of nanosheets collected in the fractions after liquid cascade centrifugation, a known volume of the higher mass fractions isolated at lower centrifugal acceleration were filtered onto alumina membranes, washed and the membranes weighed to determine the concentration. For the fractions of small/thin nanosheets isolated above 6k *g*, this was now feasible due to the low mass. To nonetheless estimate the yield, extinction and absorbance spectra were recorded and

converted to the respective coefficient spectra (Figure 2SA-B). In spite of some changes in spectral shape and intensity, both extinction (Figure S2A) and absorbance (Figure S2B) coefficients were empirically found to be invariant at specific wavelength, i.e. 265 nm in extinction and 260 nm in absorbance. The corresponding coefficients ε_{265nm}=27.7 $Lg^{-1}cm^{-1}$ and α_{260nm}=17.3 $Lg^{-1}cm^{-1}$ were used to calculate the concentration/yield of the low mass fractions. Corresponding extinction and absorbance spectra are shown in figure S2C-D. The yields are tabulated in table S1. The combined yield of all nanosheet fractions is 8%. The procedure of determining nanosheet yield from optical spectra accurately is outlined in more detail for the pyridine-based polymer **2** in section III.6 including a discussion of size-dependent changes of the optical spectra.

While the spectra will not be discussed in more detail, we note that a characteristic peak of the pyrylium-based 2D polymer **1** is observed at 645 nm in the absorbance spectra. This peak will be used later on to trace the presence of polymer **1** after post-polymerization modification to **2** and subsequent exfoliation and size selection (section III.11).

Table S1: Yield of size-selected fractions of the pyrylium-based 2D polymer **1** after exfoliation in aqueous sodium cholate

**Sample**	**Yield**
0.4-1k *g*	2.2 %
1-3k *g*	4.7 %
3-6k *g*	0.87 %
6-30k *g*	0.10 %
30-70k *g*	0.07 %
**Total**	**8%**

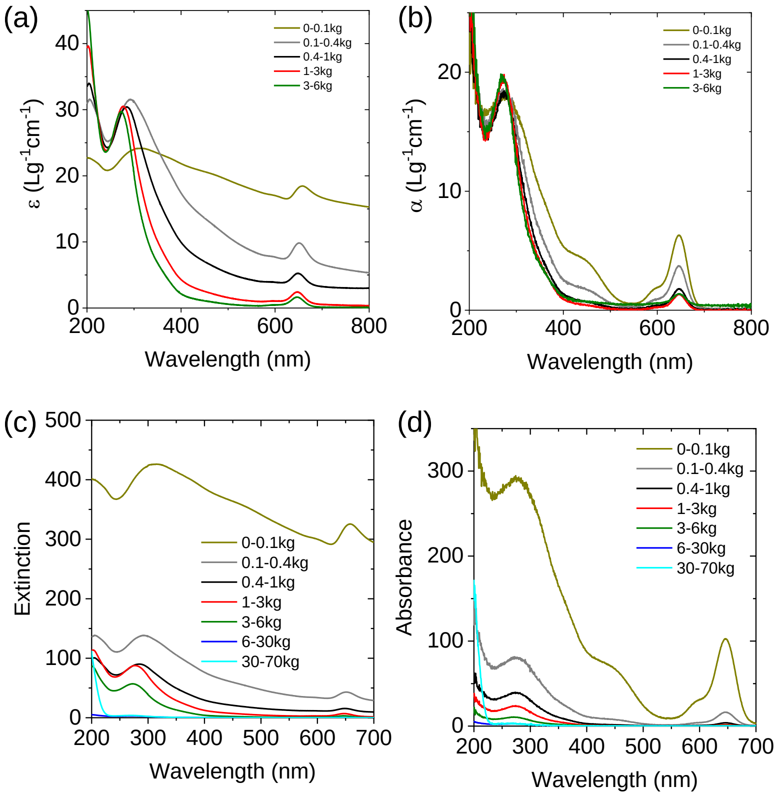

Figure S2: Extinction and absorbance spectra of size-selected fractions of the pyrylium-based 2D polymer **1** after exfoliation in aqueous sodium cholate. a) Extinction coefficient spectra of fractions isolated at low centrifugal accelerations. In these samples, the mass was sufficient for gravimetric determination. b) Absorbance coefficient spectra of the samples in b). c-d) Extinction (c) and absorbance (d) spectra of all fractions. Nanosheet concentration and yield was calculated from these spectra for samples isolated at higher centrifugal acceleration with knowledge of the size independent coefficients at 265 nm (extinction) and 260 nm (absorbance).

II.3 Transmission electron microscopy

Figure S3 shows the SAED patterns of the pyrylium-based polymer **1** after exfoliation in aqueous sodium cholate.A loss in crystallinity has been obeserved.

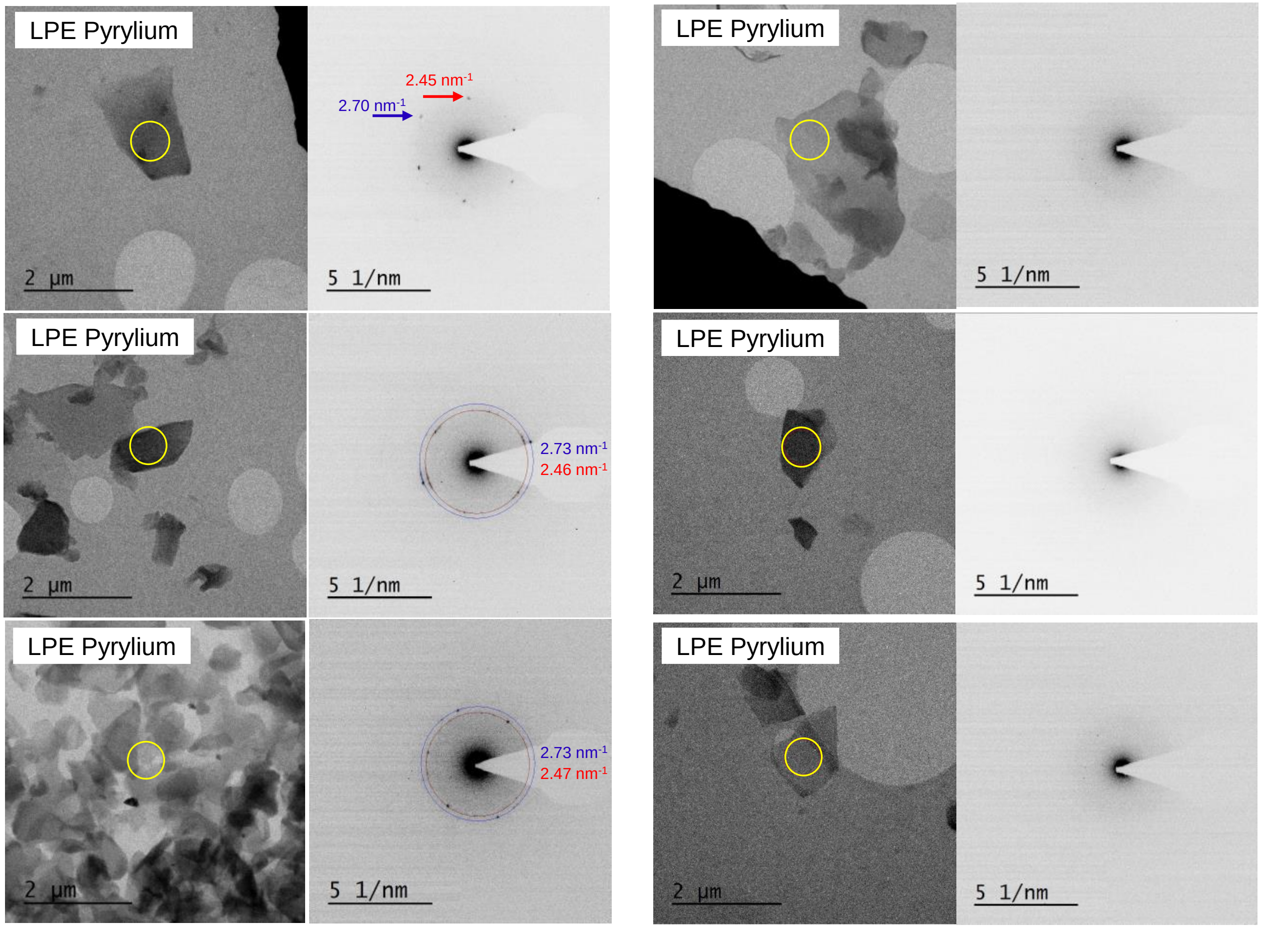

Figure S3: Bright-field TEM images and corresponding SAED patterns (electron dose: 0.2 $e^-/Å^2$) of the pyrylium-based 2D polymer **1** after exfoliation in aqueous sodium cholate. While the presence of nanosheets with characteristic shapes similar to the objects in the AFM images are observed, the SAED patterns show that most of the sheets are completely amorphous. In some cases, reflections are observed at 2.73 nm^{-1} and 2.47nm^{-1}, which are inconsistent with the expected diffraction pattern shown in the main text (i.e., Fig. 2b) .

III. POST-POLYMERIZATION MODIFICATION

III.1 Procedures for the synthesis of model compounds and the model reactions

III.1.1 Synthesis of 2,6-di-tert-butyl-4-(4-methylstyryl)pyrylium tetrafluoroborate A [6]

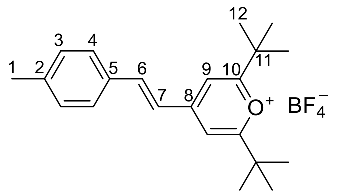

In a 100 mL round-bottom flask equipped with a magnetic stir bar, 1.50 g of 2,6-di-*tert*-butyl-4-methylpyrylium tetrafluoroborate (5.10 mmol, 1.00 eq.), 674 mg of 4-methylbenzaldehyde (5.61 mmol, 1.10 eq.) and 45 mL acetic acid were added. The suspension was refluxed at 130 °C for 14 h and cooled to room temperature. The crude reaction product was poured in 150 mL of diethyl ether and kept in the fridge for 2 h. After filtration over a glass frit and subsequent washing with diethyl ether (3 × 50 mL) the product was recrystallized from EtOH:Et_2O (1:1 v/v) via solvent evaporation. The title compound was obtained as orange crystals (1.89 g, 94%) – ^{1}H NMR ($CDCl_3$, 300 MHz) δ = 1.47 (s, 18 H, *t*Bu-C*H*$_3$), 2.35 (s, 3 H, Ar-C*H*$_3$), 7.16 (d, $^3J_{HH}$ = 8.0 Hz, 2 H, Ar-*H*), 7.43 (d, $^3J_{HH}$ = 16.0 Hz, 1 H, Vinyl-*H*), 7.76 (d, $^3J_{HH}$ = 8.1 Hz, 2 H, Ar-*H*), 7.90 (s, 2 H, Pyr-*H*), 8.31 (d, $^3J_{HH}$ = 16.0 Hz, 1 H, Vinyl-*H*) ppm – ^{13}C NMR ($CDCl_3$, 76 MHz) δ = 183.90 (C10), 165.73 (C8), 152.17 (C6), 144.23 (C2), 132.16 (C5), 130.96 (C4), 130.17 (C3), 122.47 (C7), 113.96 (C9), 38.74 (C11), 28.21 (C12), 22.00 (C1) ppm – HRMS (MALDI) m/z 309.22112 (calc. M^+ 309.22129) – IR (ATR): ṽ = 2968 (w), 2934 (w), 2916 (w), 2873 (w), 1641 (w), 1614 (m), 1587 (vs), 1566 (s), 1523 (s), 1468 (m), 1468 (m), 1424 (m), 1369 (m), 1346 (m), 1333 (m), 1312 (m), 1285 (w), 1268 (w), 1245 (w), 1218 (w), 1207 (w), 1186 (m), 1114 (m), 1046 (vs), 1033 (vs), 987 (s), 945 (s), 910 (m), 897 (m), 870 (m), 816 (s), 775 (w), 755 (w), 711 (w), 639 (w), 639 (w), 520 (w), 494 (m) cm^{-1}.

The crystal structures of **A** and **B** (see following section) were already investigated by Buchholz & Enkelmann,[6] but a re-examination was necessary to confirm the success of the synthesis. The crystallization conditions were applied as reported, but measurement parameters (100 K instead of room temperature) differed from the original work of Buchholz & Enkelmann. The found crystal structures differ in three aspects and the following description applies to the unreacted and dimerized crystals. First, there are significant differences in the space groups and unit cells. The space groups of the original structures were found to be *Cc* while in this study the space groups were found to be $P2_1/n$. This is accompanied by reduced unit cell parameters. Second, dimerizable pairs of **A** and dimers **B** are similarly arranged relative to each other in both studies but the relative arrangement of such pairs with respect to other pairs is different. In the original study, two pairs can be described in a head-head positioning, but in our current study, they were found in a tail-head positioning. Finally, a single pyrylium arm was found to be much more disordered than in the original paper. In addition to the already known disordered *tert*-butyl groups, the olefin bonds branch of into two different directions, similar as in comparable molecules found in literature.[1, 7] For post-dimerization modifications, the dimerized crystal **C** was recrystallized which led to another change in the packing where all dimmer pairs are arranged quasi parallel to each other.

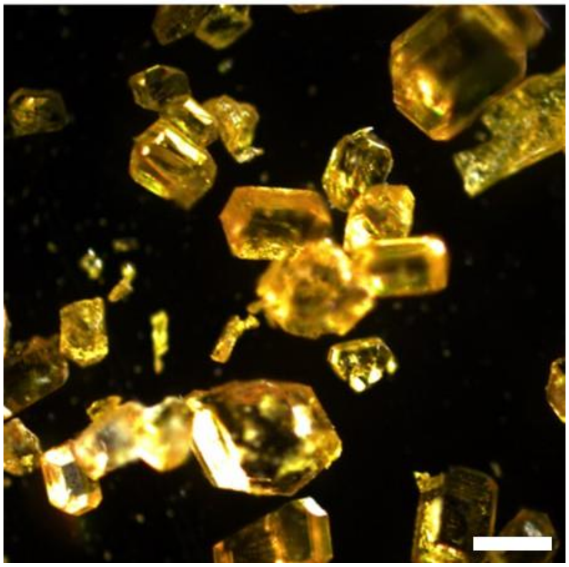

Figure S4a) OM darkfield image of single crystalline **A** after recrystallization. Scale bar: 200 µm.

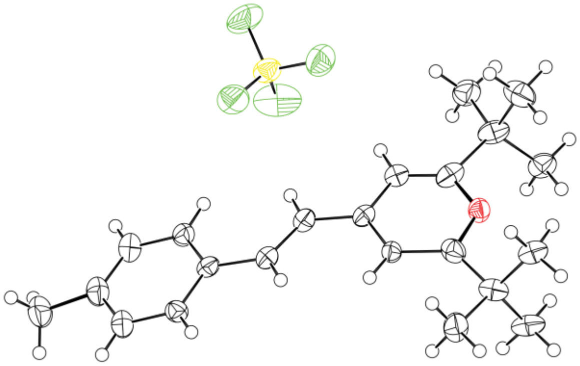

Figure S4b) ORTEP representation of **A** derived from sc-XRD analysis.

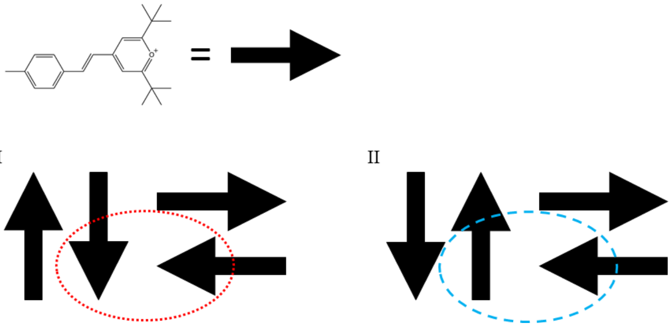

Figure S4c) Differing relative orientations of multiple pyrylium pairs of **A** found by Buchholz & Enkelmann with a head-head arrangement (red, dotted cricle) and this study with a tail-head arrangement (blue, dashed circle).

Crystallographic data of A

Identification code	sc020717_1_1 (CSD entry requested)
Empirical formula	$C_{22}H_{29}BF_4O$
Formula weight	396.26
Temperature/K	100.0(1)
Crystal system	monoclinic
Space group	$P2_1/n$
a/Å	11.1093(2)
b/Å	15.7624(2)
c/Å	12.71630(10)
α/°	90
β/°	101.8500(10)
γ/°	90
Volume/Å^3	2179.29(5)
Z	4
ρ_{calc}g/cm^3	1.208
μ/mm^{-1}	0.796
F(000)	840.0
Crystal size/mm^3	0.193 × 0.11 × 0.094
Radiation	CuKα (λ = 1.54184)
2Θ range for data collection/°	9.054 to 159.404
Index ranges	$-13 \le h \le 13$, $-19 \le k \le 20$, $-16 \le l \le 16$
Reflections collected	55916
Independent reflections	4682 [R_{int} = 0.0464, R_{sigma} = 0.0193]
Data/restraints/parameters	4682/565/466
Goodness-of-fit on F^2	1.047
Final R indexes [I>=2σ (I)]	R_1 = 0.0460, wR_2 = 0.1306
Final R indexes [all data]	R_1 = 0.0534, wR_2 = 0.1363
Largest diff. peak/hole / e Å^{-3}	0.41/-0.21

III.1.2 Synthesis of 4,4'-(2,4-di-p-tolylcyclobutane-1,3-diyl)bis(2,6-di-tert-butylpyrylium) bis(tetrafluoroborate) B

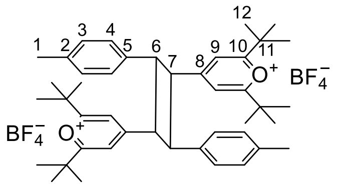

Single crystals of **A** were put in a 4 mL vial and sealed with a Teflon cap. The vial was placed in a custom-built 530 nm photo reactor in a fridge at 4 °C and irradiated overnight. The photoreaction gave yellow crystals of 4,4'-(2,4-di-*p*-tolylcyclobutane-1,3-diyl)bis(2,6-di-*tert*-butylpyrylium) bis(tetrafluoroborate) **B** in quantitative yield – ^{1}H NMR (TFA-*d*, 300 MHz) δ = 1.37 (s, 36 H, *t*Bu-C*H*$_3$), 2.24 (s, 6 H, Ar-C*H*$_3$), 5.07 (s, 4 H, cyclobutane-*H*), 7.14 (d, $^{3}J_{HH}$ = 7.7 Hz, 4 H, Ar-*H*), 7.19 (d, $^{3}J_{HH}$ = 7.5 Hz, 4 H, Ar-*H*), 7.64 (s, 4 H, Pyr-*H*) – ^{13}C NMR (TFA-*d*, 76 MHz) δ = 189.66 (C10), 179.10 (C8), 131.69 (C2), 135.07 (C5), 132.27 (C3), 130.05 (C4), 120.66 (C9), 51.00 (C7), 49.13 (C6), 41.21 (C11), 28.87 (C12), 21.45 (C1) ppm – HRMS (MALDI) m/z 705.44687 (calc. M$^+$ 705.44605) – IR (ATR): ṽ = 2974 (w), 2939 (vw), 2916 (vw), 2875 (vw), 1619 (m), 1523 (m), 1483 (w), 1465 (w), 1448 (w), 1424 (vw), 1369 (w), 1315 (vw), 1240 (w), 1218 (vw), 1194 (w), 1047 (s), 1029 (vs), 967 (w), 956 (w), 948 (m), 935 (w), 914 (m), 869 (w), 852 (w), 815 (m), 799 (w), 774 (w), 683 (vw), 609 (vw), 594 (vw), 532 (w), 521 (m) cm^{-1}.

For larger quantities, the single crystals were suspended in cyclohexane and irradiated in the same array under vigorous stirring. After removal of the solvent by rotary evaporation and subsequent drying under vacuum, the product is obtained as light yellow powder in quantitative yield.

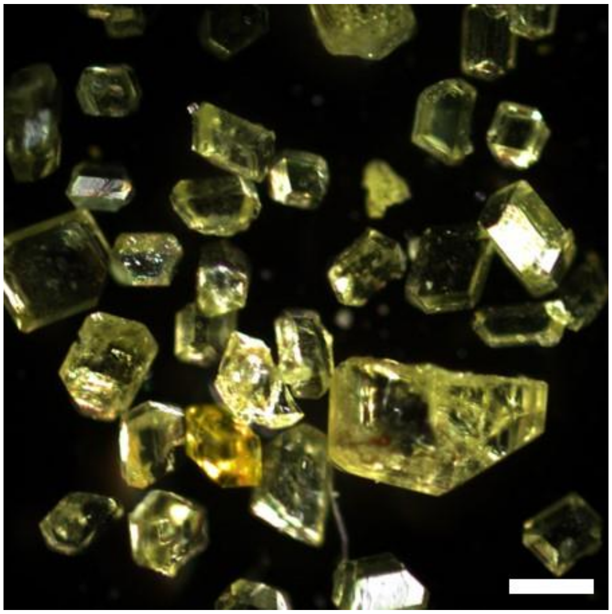

Figure S5a. OM darkfield image of single crystalline **B**. Scale bar: 200 μm.

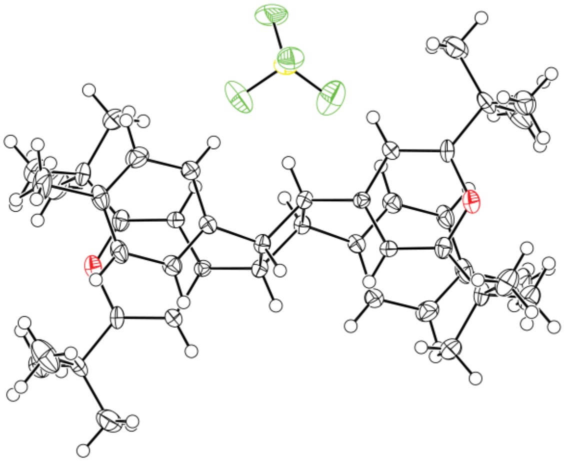

Figure S5b. ORTEP representation of **B** derived from sc-XRD analysis.

Crystallographic data of B

Identification code	sc120717_1_1 (CSD entry requested)
Empirical formula	$C_{44}H_{58}N_2$
Formula weight	614.92
Temperature/K	100.0(2)
Crystal system	monoclinic
Space group	$P2_1/c$
a/Å	14.6883(2)
b/Å	11.00280(10)
c/Å	12.06160(10)
α/°	90
β/°	106.0040(10)
γ/°	90
Volume/Å^3	1873.75(4)
Z	2
ρ_{calc}g/cm^3	1.090
μ/mm^{-1}	0.463
F(000)	672.0
Crystal size/mm^3	0.086 × 0.075 × 0.043
Radiation	CuKα (λ = 1.54184)
2Θ range for data collection/°	6.26 to 158.118
Index ranges	$-18 \le h \le 18$, $-14 \le k \le 13$, $-15 \le l \le 15$
Reflections collected	70767
Independent reflections	4028 [R_{int} = 0.0525, R_{sigma} = 0.0182]
Data/restraints/parameters	4028/0/215
Goodness-of-fit on F^2	1.075
Final R indexes [I>=2σ (I)]	R_1 = 0.0402, wR_2 = 0.1017
Final R indexes [all data]	R_1 = 0.0462, wR_2 = 0.1052
Largest diff. peak/hole / e Å^{-3}	0.26/-0.20

III.1.3 Synthesis of 4,4'-(2,4-di-p-tolylcyclobutane-1,3-diyl)bis(2,6-di-tert-butylpyridine) C

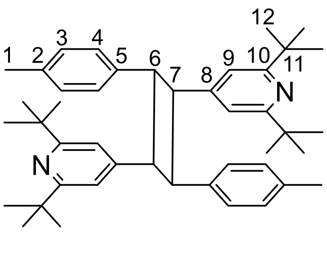

Method A: A suspension of 85 mg of single crystalline **B** (0.107 mmol, 1.00 eq.) in 3 mL 30 wt% ammonia water was vigorously stirred at 60 °C for 2 h. The suspended solid was filtered via a glass frit and washed with H_2O (3 × 10 mL). The title compound was obtained as white crystals (63 mg, 96%) after recrystallization from EtOH.

Method B: A 4 mL vial with 20 mg of single crystalline **B** was submerged in a 25 mL vial filled with 3 mL of 30 wt% ammonia water and sealed with a screwcap. After 24 h the crystals were taken out, washed with H_2O (2 × 10 mL) and EtOH (2 × 10 mL). The title compound was obtained as white crystals (14 mg, 90%) which were used for sc-XRD structure analysis after recrystallization from EtOH – ^{1}H NMR ($CDCl_3$, 300 MHz) δ = 1.18 (s, 36 H, *t*Bu-CH_3), 2.22 (s, 6 H, Ar-CH_3), 4.34 (m, 4 H, cyclobutane-*H*), 6.70 (s, 4 H, Pyr-*H*), 6.95 (s, 8 H, Ar-*H*) ppm – ^{13}C NMR (TFA-*d*, 76 MHz) δ = 166.47 (C10), 165.13 (C8), 141.54 (C2), 135.77 (C5), 132.33 (C3), 129.85 (C4), 124.03 (C9), 49.99 (C7), 49.30 (C6), 38.72 (C11), 29.68 (C12), 21.50 (C1) ppm – HRMS (MALDI) m/z 615.46719 (calc. MH^+ 615.46728) – IR (ATR): ṽ = 2956 (s), 2903 (m), 2865 (m), 1595 (vs), 1563 (s), 1513 (m), 1479 (m), 1457 (m), 1421 (m), 1390 (w), 1357 (s), 1255 (m), 1230 (w), 1204 (w), 1186 (w), 1167 (w), 1135 (w), 1031 (w), 1019 (w), 935 (w), 927 (w), 901 (w), 866 (m), 853 (m), 809 (s), 772 (m), 684 (w), 567 (w), 523 (s) cm^{-1}.

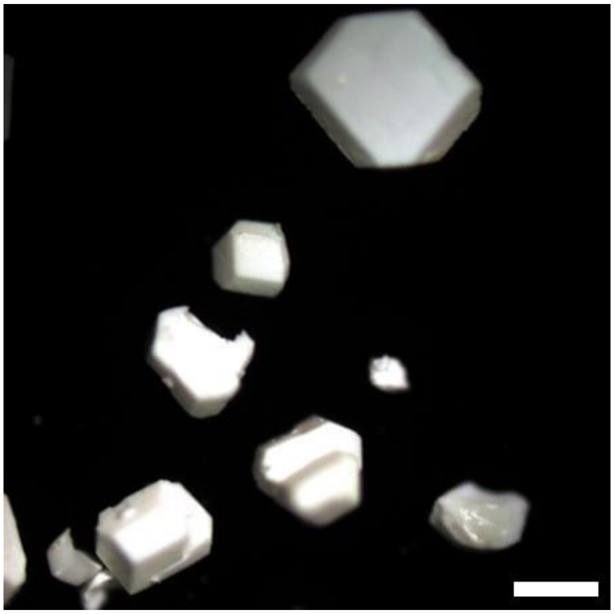

Figure S61a) OM darkfield image of **C** *before* recrystallization. Transparency of crystals is lost during chemical reaction. Scale bar: 200 μm.

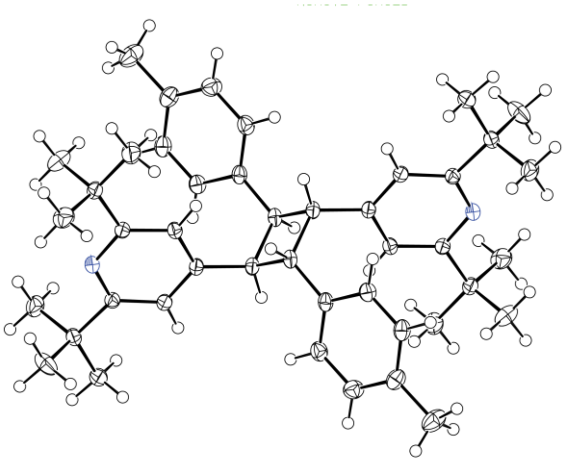

Figure S6b) ORTEP image of **C** acquired by sc-XRD analysis *after* recrystallization.

Crystallographic data of C

Identification code	sc120717_1_1 (CSD entry requested)
Empirical formula	$C_{44}H_{58}N_2$
Formula weight	614.92
Temperature [K]	100.0(2)
Crystal system	monoclinic
Space group	$P2_1/c$
a [Å]	14.6883(2)
b [Å]	11.00280(10)
c [Å]	12.06160(10)
α [°]	90
β [°]	106.0040(10)
γ [°]	90
Volume [Å^3]	1873.75(4)
Z	2
ρ_{calc} [g/cm^3]	1.090
μ [mm^{-1}]	0.463
F(000)	672.0
Crystal size [mm^3]	0.086 × 0.075 × 0.043
Radiation	CuKα (λ = 1.54184)
2θ range for data collection [°]	6.26 to 158.118
Index ranges	$-18 \le h \le 18$, $-14 \le k \le 13$, $-15 \le l \le 15$
Reflections collected	70767
Independent reflections	4028 [R_{int} = 0.0525, R_{sigma} = 0.0182]
Data/restraints/parameters	4028/0/215
Goodness-of-fit on F^2	1.075
R_{int}	0.0525
Final R indexes (I>=2σ (I))	R_1 = 0.0402, wR_2 = 0.1017
Final R indexes (all data)	R_1 = 0.0462, wR_2 = 0.1052
Largest diff. peak/hole [e Å^{-3}]	0.26/-0.20

III.2 Details of model studies

The investigations concerning the post-polymerization of 2D polymer **1** started with a model reaction. This served to develop the synthetic methodology in a simpler case and to generate spectroscopic data needed later for reference purposes.

The styryl pyrylium salt **A** was reacted to the corresponding dimer **B** in the single crystal according to Scheme S1. After exploring a few conditions for how to expose the crystals of **B** to ammonia (see two methods for the synthesis of **C** in 0), it was decided to concentrate on a heterogeneous reaction in which the crystals were exposed to in a sealed vial at room temperature. The products of this reaction were analyzed by ^{1}H and ^{13}C NMR spectroscopy, mass spectrometry and IR spectroscopy. The whole sequence from **A** to **C** was also investigated by optical microscopy (Figures S4 – S6a) and sc-XRD (Figure S4-6b).

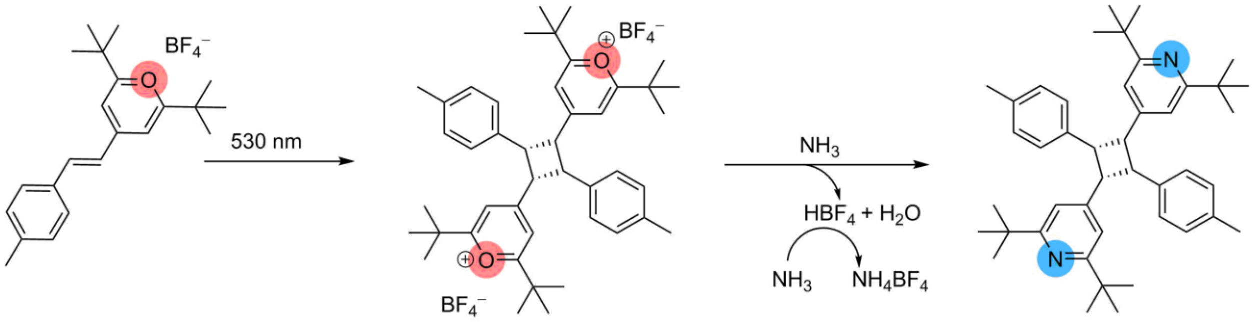

A **B** **C**

Scheme S1. Synthesis of the model compounds **A – C**.

The following results were obtained:

1. Dimer **B** has the expected stereochemistry, directly resulting from the packing of compound **A**.
2. The conversion of **B** to **C** does not proceed in scsc fashion. Changing typical factors such as ammonia concentration and temperature (–20 °C instead of room temperature) always resulted in fractured crystals, which did not sufficiently scatter anymore for sc-XRD. This is the reason why the crystals of **C** in the optical micrograph appear opaque (Figure S4). For obtaining the crystal structure, compound **C** was recrystallized.
3. The reaction of **B** to **C** proceeded virtually quantitative, as the yield of recrystallized **C** amounted to >95%.

Two aspects were of further interest. They concern the changes in the ^{13}C NMR and the IR spectra caused by the transformation of **B** to **C**, thus by converting pyrylium into pyridine. Figure S4 displays the shift differences and Figure S5 the IR spectra associated with this transformation.

The ^{13}C NMR signals of the α- and γ-C atoms exhibit a significant high field shift when going from pyrylium to pyridine. These shift changes amount to approximately 15 and >20 ppm and cause both C-atoms in the pyridine derivative **C** to appear almost isochronically at about δ = 166 ppm (Figure S7). This is an interesting benchmark when later considering the post-polymerization modification. Thus, also here one would expect the signals to merge at higher field.

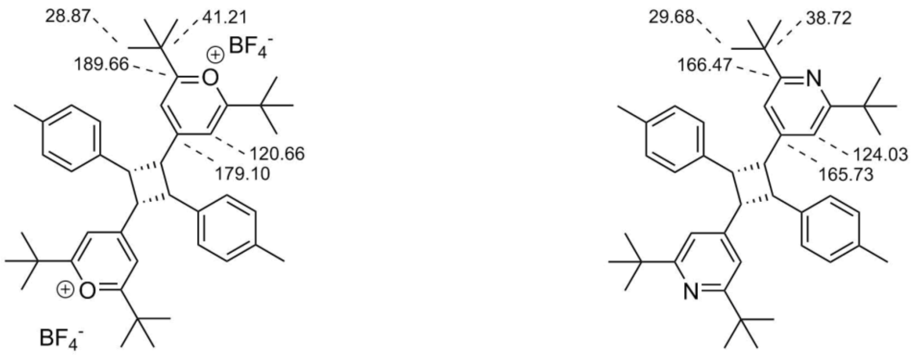

B **C**

Figure S7. Relevant ^{13}C NMR chemical shifts in ppm of compounds **B** and **C**. Note that the signals of the α- and the γ-C-atoms of **B** are shifted high field to almost the same shift value in compound **C**.

The changes in the IR spectra caused by the exposure of the crystals of **B** to ammonia are reflected by Figure S8. To facilitate the discussion, this figure also contains the IR spectrum of neat $NH_4^+BF_4^-$ (yellow), where the signals centered at approximately 3330 cm^{-1} and 1425 cm^{-1} are assigned to vibrations associated with NH_4+ and the signal centered at approximately 1020 cm^{-1} to the vibrations of the BF_4^- group. The spectrum of grinded crystals of **B** (blue) shows a strong absorption in the region characteristic for BF_4^- but no absorptions due to NH_4^+, which is in line with the expectation that the pyrylium cation acts as the counter ion for BF_4^-. As the crystals of **B** are exposed to ammonia, not only the pyridines of **C** are formed but also NH_4^+ ions, which counter

balance BF_4^-. Consequently, all three absorptions required for $NH_4^+BF_4^-$ are now visible (blue spectrum). The last spectrum in the sequence (red) refers to a purified sample of **C**. It shows the interesting fact that the formed $NH_4^+BF_4^-$ can actually be removed from the ground crystals by briefly shaking them in an ethanol/water mixture (1:1 v/v).

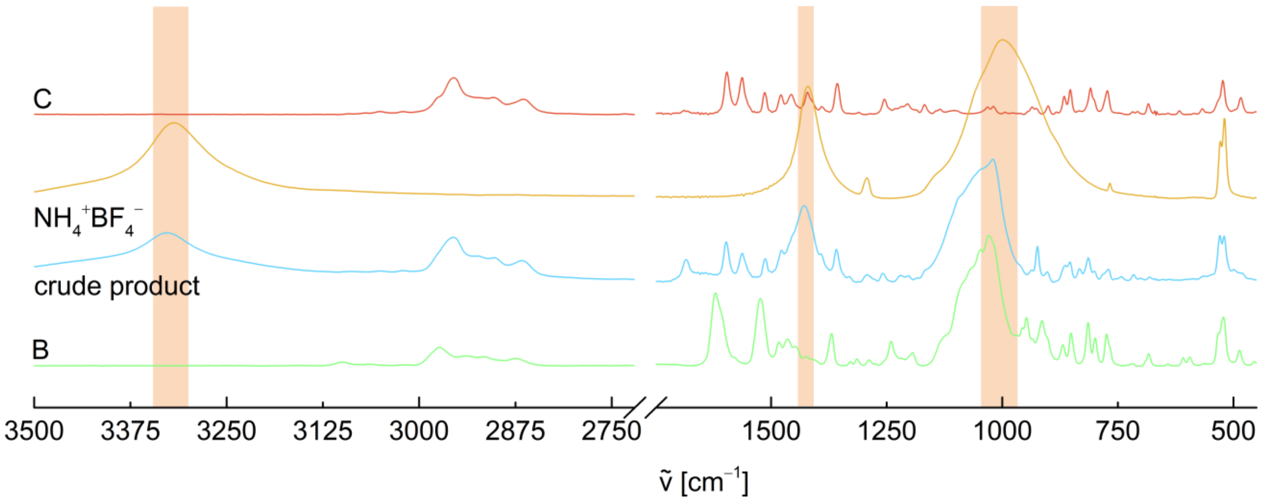

Figure S8. ATR-IR spectra on the conversion of model pyrylium salt **B** into its pyridine derivative **C**. Green: starting material **B**; blue: crude product **C**; brown: authentic $NH_4^+BF_4^-$; red: purified product **C**.

The pyrylium model salt **B** was also directly compared with its pyridine derivative **C** to find a characteristic difference between both entities in IR spectroscopy. Such a difference could then later be used when addressing the crucial question concerning the efficiency of the same transformation applied to 2D polymer **1**.

For the two parent entities, the unsubstituted pyrylium ion and pyridine, Balaban has reported that the C-C vibration at 1620 cm^{-1} in the former shifts to 1583 cm^{-1} in the latter.[8] These vibrations

do not seem to change much considering the fact that the IR spectra of the more elaborate structures **B** and **C** show signals at 1620 cm^{-1} and 1595 cm^{-1}, respectively (Figure S9).

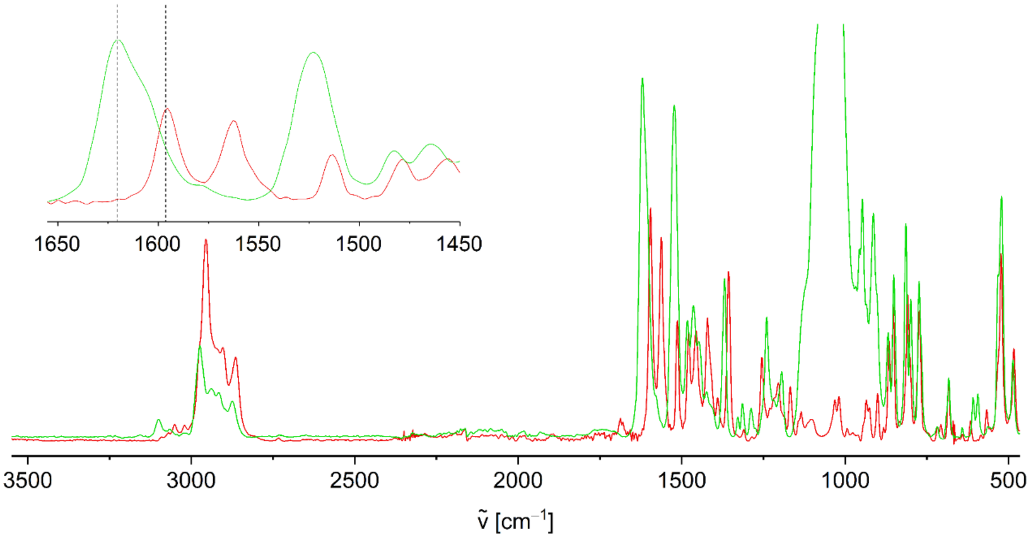

Figure S9. IR-spectroscopical comparison of model salt **B** (green) with compound **C** (red). The signals at 1620 cm^{-1} and 1593 cm^{-1} are assigned to pyrylium and pyridine vibrations, respectively, based on literature assignments.[8] Spectra are not normalized because of large reversing intensities in the regions 2800 – 3000 cm^{-1} and 500 – 1700 cm^{-1}.

The crystal structure of compound **B** does not contain pores. These obvious transportation paths are thus not available. A brief look was therefore taken into free and solvent accessible volumes as well as into displacements within the single crystals of **B**. The two kinds of volumes are measures for how densely a structure is packed and the level of displacements mirrors possible dynamics within the crystal.

Applying the Connolly surface (radius 1 Å)[9] a free volume of more than 10% is obtained. The solvent accessible volume (Program Platon, Version 230318),[10] amounts to approximately 100 Å^3 per elementary cell, a number referring to approximately 5% of the cell volume. This volume is

concentrated in four main voids, which are not directly connected (Figure S10). Both the di-cation **B** and the tetrafluoroborate anion exhibit considerable disorder of in the structure. It is thus reasonable to assume that there is mobility within the crystal by which the small ammonia molecules could be pushed from one solvent accessible volume void into another by the moving ions. This rationalizes how ammonia actually manages to reach all reactive sites.

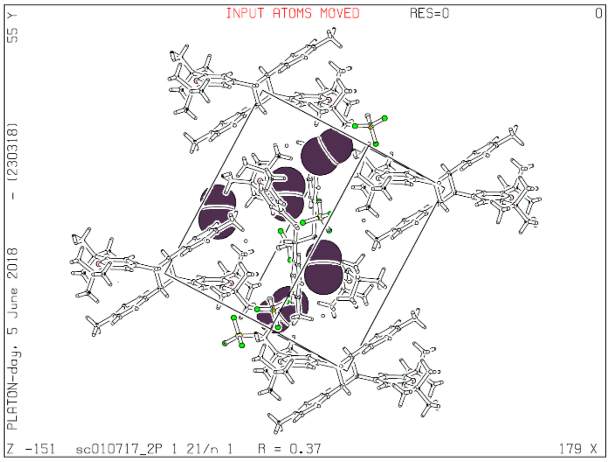

Figure S10. Representation of the solvent accessible volume in the crystal structure of the tetrafluoroborate salt of **B**. Together with the expected high dynamics in the structure caused by considerable displacements of both ions, it should be possible for a small molecule such as ammonia to reach every site within the crystal.

III.3 Details of the post-polymerization modification of 2D polymer 1

The post-polymerization conversion of 2D polymer **1** is of importance for reasons including:

1. It represents the first case in which a 2D polymer is chemically modified in the plane. This way, an existing 2D polymer is converted into a chemically rather different 2D polymer without having to go through the synthesis of carbon skeleton of the new polymer. This not only saves work but increases the versatility of 2D polymer **1**.
2. It allows to render a sheet-like polyelectrolyte, the 2D polymer **1** with its three positive charges per through-pore in the ABC stack, into a neutral 2D polymer with the non-charged pyridines at the pore rims rather than pyrylium ions. Considering the differences between linear polyelectrolytes and their non-charged counter parts, one can expect this modification to result in severe property changes e.g. regarding the solubility and aggregation behavior but also concerning the through pore separation characteristics.
3. It could impact the exfoliation behavior because Coulombic interactions between the individual 2D polymer sheets within a stack of sheets are replaced by van der Waals interactions.

The conversion of 2D polymer **1** (Figure 2) was attempted under the conditions that were optimized in the model reaction described in the previous chapter. We used the same sample of 2DP **1** whose synthesis we previously reported.[1] The crystals typically had sizes between 50 – 200 µm measuring the longest side. The reaction was carried out by placing a few dry single crystals **1** in a 4 mL vial. This vial in turn was submerged in a 25 mL vial containing a bath of 3 mL conc. ammonia water and sealed airtight with a screw cap.

Within a matter of seconds, the initially yellow, transparent crystals turned slightly greenish and lost their transparency (Figure 3c). The exposure was stopped after 60 minutes. Obviously, a reaction had taken place and the loss of transparency indicated that the single crystalline order was likely destroyed. While this may not astound considering the complexity of the structural changes involved, it complicated the matter of conversion determination considerably (see below).

When having a closer look into the packing of 2D polymer **1**, one realizes a rather different situation from that of model salt **B**. Whereas the latter did not show pores, the former not only has through pores with an approximate diameter of 5 Å but also one of the α-C-atoms of the pyrylium ion moiety is pointing towards the pore 'wall' (Figures 3a,b). This suggests, that the reactive unit is accessible by reagents small enough to penetrate the pore and. In addition, reactivity is anticipated because the distance between the pyrylium ions is 1.62 nm. This distance is associated with enough space for the ammonia molecules to attack the α-C-atoms. Figures 3a and b illustrate this, by showing the top view of the pore in a single layer and the through pore in ABC-stacked layers. In conclusion, the packing of 2D polymer **1** suggests facile accessibility of all pyrylium ion moieties within the single crystal and high reactivity. Together with the observed instantaneous reaction of the crystals at room temperature, this nourished hopes for a high reaction conversion.

The modification was analysed by CP/MAS ^{13}C NMR and ATR-IR-spectroscopy as well as by PXRD. As indicated above, sc-XRD could unfortunately not be applied anymore because the losses of crystallinity caused by the severe structural changes. The CP/MAS ^{13}C NMR spectra of starting material and product reveal two main differences (Figure S11). They concern broadness of signals and disappearance/appearance of signals.

Already by visual inspection it becomes clear that the full width at half maximum of the product signals is much larger than of the starting material. This reflects the decreased crystallinity and doers not facilitate signal assignment. Regarding the chemical shift changes of signals it is nevertheless striking that the 2D polymer **1** shows three downfield signals at δ = 186, 184 and 179 ppm, while the product has only one such (broad) signal centred at δ = 167 ppm. Based on the model studies above, the two signals at δ = 186 and 184 ppm were assigned to the pyrylium α-C-atoms. These atoms appear as two signals because of symmetry-breaking in the single crystal (Figure S11). The signal at 179 ppm is assigned to the pyrylium γ-C-atom. These three signals disappeared altogether during the exposure of 2D polymer **1** to ammonia as is required for the proposed reaction. Furthermore, the newly formed signal at δ = 167 ppm is right in the range expected for the α- and γ-C-atoms of pyridines and thus constitutes clear support for the transformation shown in Figure 2a.

While it is thus clear that the proposed reaction has taken place and 2D polymer **2** has actually formed, the conversion to which this has happened cannot be determined with high fidelity just based on these NMR data. The reason for that lies in the relatively low signal-to-noise ratio often encountered in CP/MAS NMR spectroscopy. From the full disappearance of the three downfield signals of 2D polymer **1** one may be inclined to propose complete reaction. However, given the signal-to-noise ratio it appears safer to propose an NMR-conversion of beyond 90%.

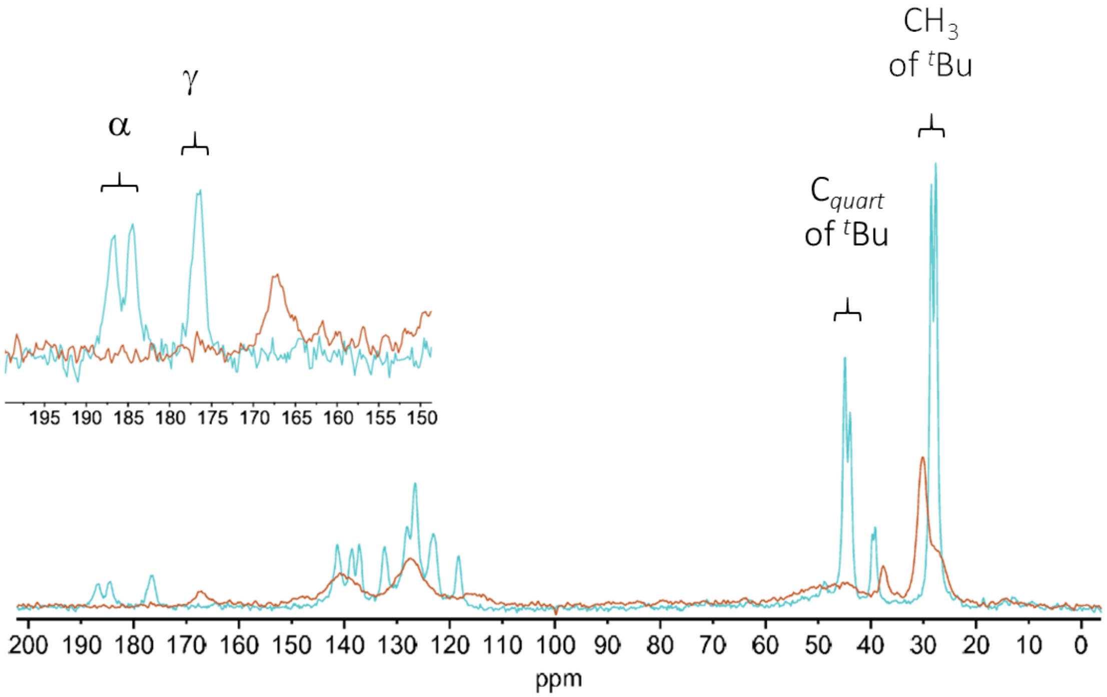

Figure S11. CP/MAS ^{13}C-NMR spectra (2.5 mm, 20 kHz) of single crystalline 2D polymer **1** (cyan) and of the anticipated 2D polymer **2** (orange) after post-polymerization modification. The decreased crystallinity associated with this process shows in line broadening. The insert concerns the region with the most important changes concerning the proposed structural modification. The two signals due to the *tert*-butyl groups in **1** are split due to desymmetrization in the crystal packing. For more explanations, see text.

This transformation of two 2D polymers into one another was also investigated by IR spectroscopy. Particular attention was placed on the aspect whether the two absorptions at 1620 cm^{-1} and 1593 cm^{-1} of the model compounds discussed in Figure S9 would be similar. As the spectra in Figure S12 show, signals at 1620 cm^{-1} and 1595 cm^{-1} do in fact appear, which are shifted negligibly compared to the models. Thus, in line with the NMR spectroscopical result, also IR spectroscopy provides clear evidence for the post-polymerization to have occurred.

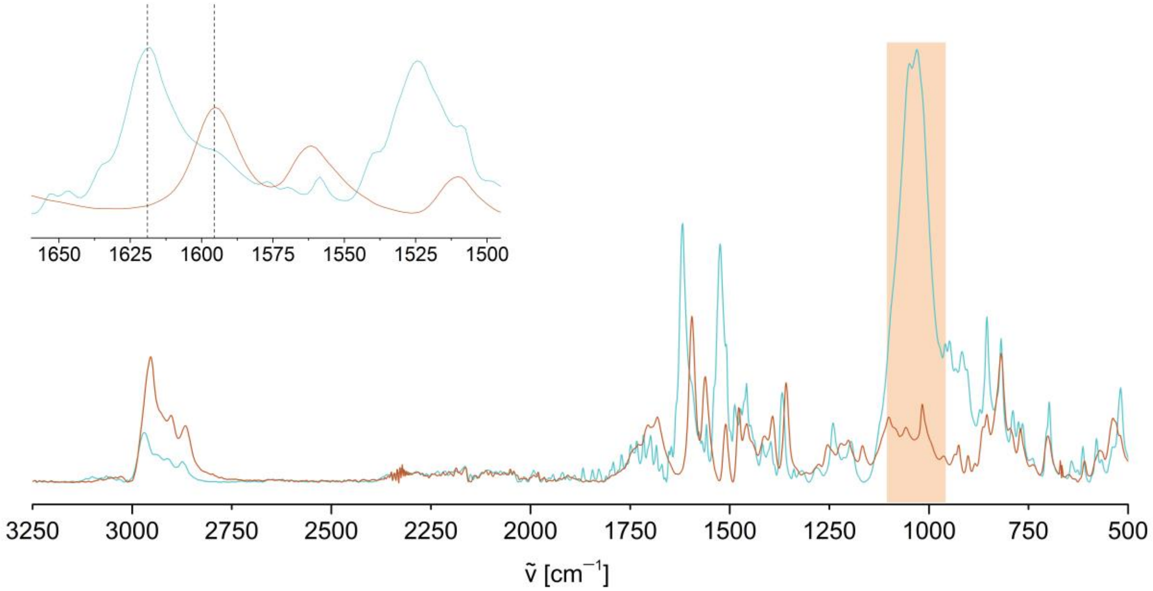

Figure S12. IR spectral evidence for the post-polymerization modification of 2D polymer **1** (cyan) into 2D polymer **2** (orange). The inset shows the signal at 1620 cm^{-1} for 2D polymer **1** and at 1595 cm^{-1} for 2D polymer **2**. Note that the intensities of the signals due to BF_4^- at approximately 1020 cm^{-1} cannot reasonably be compared because the spectra could not be normalized.

The spectra in Figure S12 provide additional insight, which concerns reaction conversion. For that purpose, we compare the two spectra in the insert of this figure in the region of approximately 1620 cm^{-1}. As can be clearly seen, there is no residual intensity of the cyan signal in the brown spectrum as would be indicated by a small shoulder. Thus, according to IR spectroscopy there is no indication for the conversion of 2D polymer **1** into 2D polymer **2** to not be complete.

Because no diffraction data could be gathered from sc-XRD experiments, a more qualitative description of the change in crystallinity was obtained with PXRD analysis. As a reference, the 2D polymer **1** was measured without further grinding. This specimen was then exposed to ammonia

gas in a sealed vial and samples were taken after 3 min and again after prolonged exposure overnight (Figure S13).

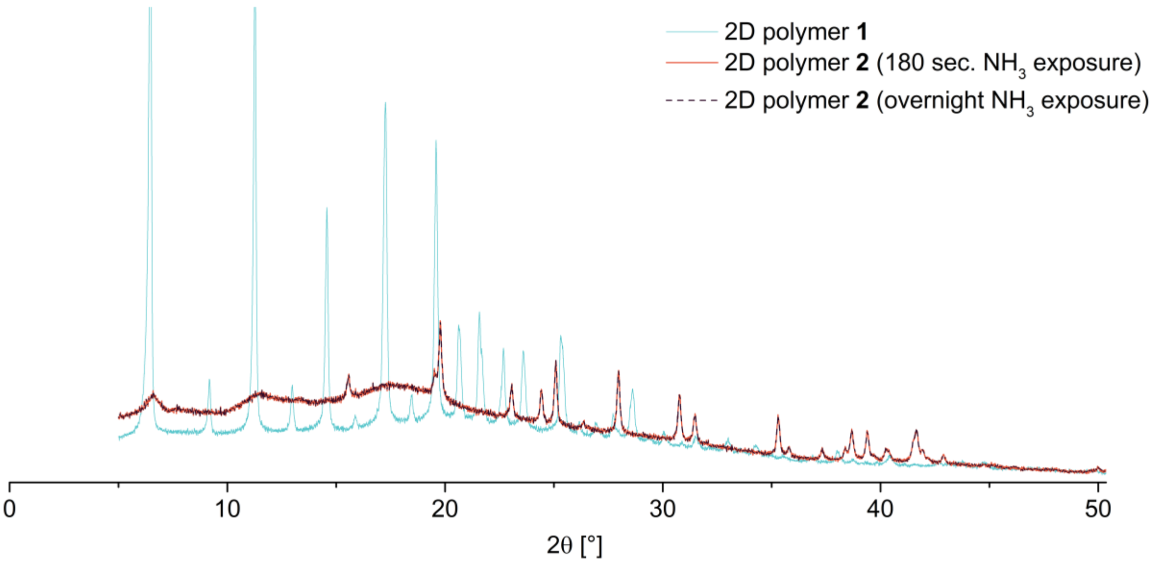

Figure S13. PXRD comparison of pyrylium 2D polymer **1** and modified pyridine 2D polymer **2**. Complete conversion was already indicated after the first sample was taken out after 3 min accompanied with a severe loss of crystallinity. Note that the diffractograms of polymer 2 overlap entirely.

While the reference **1** exhibits sharp and strong reflections, only very weak scattering was found at small angles for both samples upon differently long exposure to ammonia. This explains why the acquisition of a single crystal structure failed. Additionally, new reflections appeared in the high angle scattering regime. The patterns of both modified samples are so similar, that they cannot be distinguished from one another ion Figure S13. This hints towards full conversion already after 3 min ammonia exposure.

From a crystallographic point of view, the post-polymerization reaction replaces atoms of similar weight with each other in a point-by-point fashion, which should result in a mostly identical

diffraction pattern. Clearly, this was not the case. More likely, conformational changes in the covalent polymer framework occurred during the reaction. The smaller reflections appearing at $2\theta = 35 - 45°$ after ammonia exposure could hint to aggregation/crystallization of the side products which would result in deformation of the surrounding covalent framework. The fact that exfoliation and separation furnished fractions with high monolayer sheet content, is a strong indication for the changes in the PXRDs upon chemical conversion is not due to degradation of the crystalline sheets of 2D polymer **2**.

From the powder XRD pattern, overall lattice spacings of 0.23-0.24 nm, 0.25 nm, 0.28-0.29 nm, 0.32 nm, 0.36-0.37 nm, 0.385 nm, 0.45 nm and 0.56 nm were found. As shown below, these correspond well to the spacing found by electron diffraction of the exfoliated nanosheets.

IV. EXFOLIATION OF THE PYRIDIN-BASED POLYMER

IV.1 Exfoliation in solvents

In initial experiments, different organic solvents were screened in their capability to exfoliate the polymer **2**. For other 2D materials, it has been shown that colloidally stable dispersions of exfoliated nanosheets can be achieved when the solvent is chosen appropriately. Typically, this can be described within the framework of solution thermodynamics. Mixing of solvent and solute generally occurs when the free energy of mixing containing both an enthalpic and entropic term is negative. In molecular systems, the gain in entropy often dominates. However, in case of large solutes such as nanosheets, this term will be small and solubility is therefore governed by the enthalpy of mixing. This in turn is minimized when the solubility parameters of solvent and solute match. This approach has been widely used for other materials such as nanotubes, graphene and transition metal dichalcogenides and general expressions for 1D and 2D solutes have been derived.[11]

A number of solubility parameters exist each with their own strengths and weaknesses. Probably the simplest one is the surface energy. Hence, to obtain colloidally stable dispersions of a solute in solvents without additional stabilizers, one would have to match the surface energy of solvent and solute. The solvent surface energy, γ_S is related to the surface tension, Γ via $\Gamma = \gamma_S - TS_s$ where TS_s can be approximated as 29 mJ/m^2 for a wide range of liquids at room temperature.[12] The established models[11b, 11c] imply that, if solubility can be described within solution thermodynamics, a plot of dispersed concentration as function of solubility parameter (for example solvent surface tension) should be well described by a Gaussian envelope function.

To test whether this is the case, we have selected a range of solvents with a wide spread in surface tension (table S1), immersed 1 mg of **2** in 3 mL of the solvent and subjected the mixture to 1 h of sonication in a Branson CPX2800-E sonic bath. This approach was chosen over tip sonication due to the higher throughput. The vials were placed in hot spots of the sonic bath and the water in the tank replaced after 30 min to avoid overheating. The samples were then centrifuged at 400 *g* to remove unexfoliated material. Since the sedimentation velocity of a particle is inversely proportional to the viscosity of the solvent,[13] the centrifugation times were adjusted depending on the viscosity of the solvent as summarized in table S2. The supernatant after centrifugation was decanted and subjected to optical extinction spectroscopy. The extinction per cell length in the resonant regime will roughly correlated with the nanosheet concentration and can thus be used to express the concentration.

Optical extinction spectra are shown in figure S14. In all cases, absorbance at < 400 nm is observed that is attributed to **2** (see section II.6). Since many solvents absorb in the UV region, the entire spectrum is not always accessible. We therefore use the optical density at the lowest wavelength accessible in all solvents (285 nm) as estimate for the nanosheet concentration. The extinction spectra in figure S14a-c are grouped in different panels to avoid clutter. In all cases the water-sodium cholate system that was used for the remainder of the study is included for comparison. In figure S14a, the spectra of the best solvents are shown. Figure S14b displays the spectra of solvents that give a similar spectral shape as the sodium cholate reference, but at lower concentration. In some cases (figure S14c), additional features appear in the spectra. These can either be a manifestation of strong interaction of the solvent with **2** or an indication for decomposition.

Table S2: Tabulated values of solvent viscosity, density and surface tension along with centrifugation time used and optical densities at 285 nm (OD_{285nm}) as a measure of the concentration of **2**.

Solvent	Viscosity, η (kg m^{-1} s^{-1})	Density, ρ (kg m^{-3})	Surface Tension (mJ/m^2)	Cent. Time (h)	OD_{285nm}
NMP	1.75	1.028	40.1	3.5	1.95
DMF	0.92	0.948	37.1	1.8	1.73
CHP	11.50	1.007	43.2	23.0	1.52
GBL	1.43	1.130	46.5	2.9	1.36
H_2O-SC					0.99
Chlorobenzene	0.80	1.110	33.0	1.6	0.94
$CHCl_3$	0.56	1.489	26.6	1.1	0.79
Acetonitrile	0.39	0.786	28.5	0.8	0.51
Toluene	0.59	0.870	28.5	1.2	0.45
IPA	2.38	0.786	21.7	4.8	0.29
DMEA	2.70	0.890	51.6	5.4	0.21
THF	0.61	0.889	26.6	1.2	0.17
Formamide	3.30	1.133	57.0	6.6	0.03
H_2O	1.00	0.998	72.7	2.0	0.01

In figure S14d, the optical density at 285 nm (as measure for the nanosheet concentration) is plotted as function of solvent surface tension. We indeed find that the data can be described by a Gaussian envelope function consistent with solution thermodynamics. The peak is centered at 41 mJ/m^2 corresponding to a surface energy of 70 mJ/m^2. Note that this is very close to what is observed for other systems.[11c, 11d, 14]

Some of the solvents under study give higher dispersed concentration than the aqueous surfactant system. Notably these are *N*-methyl-2-pyrrolidone, *N*-cyclohexyl-2-pyrrolidone,

chlorobenzene and potentially DMF and γ-butyrolactone. However, the latter two give additional features of unknown origin and should likely be avoided. Since no non-toxic, low boiling point solvent was identified, we have chosen the aqueous surfactant system due to ease of handling (toxicity, boiling point, viscosity, vapor pressure etc.).

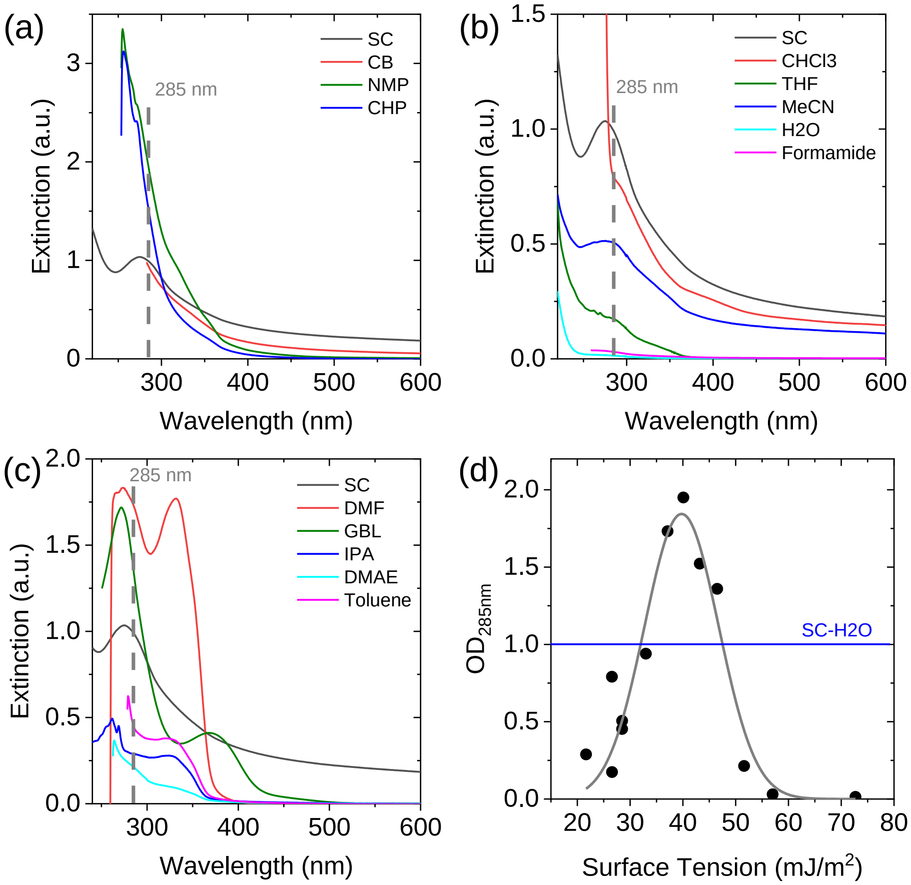

Figure S14: Optical extinction spectra of **2** dispersed in various solvents by bath sonication and centrifugation at 400 *g* to remove unexfoliated material. The spectra are arranged in groups: **a)** Good solvents giving a higher nanosheet concentration and a spectral shape similar to the water-SC reference, **b)** Solvents that give the usual spectral shape, but low concentration, **c)** Solvents that give rise to spectra with a different shape. **d)** Plot of the optical density at 285 nm indicative of the nanosheet concentration as function of solvent surface tension. Similar to other materials, the data can be described by a Gaussian envelope function with a peak centered at 41 mJ/m^2.

IV.2 Transmission electron microscopy

Figure S15 shows the SAED patterns of the pyridin-based polymer **2** after exfoliation in aqueous sodium cholate. The 0.4-1k *g* fraction (<N>=18, <L>=870 nm) was drop-cast onto Quantifoil or lacey carbon TEM grids. In contrast to the charged precursor **1**, nanosheets exhibit higher crystallinity. Different nearest reflections are found depending on the orientation of the nanosheet under the beam. Overall, we identified reflections corresponding to spacings of 0.45 nm, 0.44 nm, 0.43 nm, 0.40 nm, 0.33 nm, 0.26 nm, 0.25 nm, 0.24 nm, 0.21 nm and 0.17 nm. This is broadly consistent with the result from the powder diffratogram (section II.3) and confirms that LPE does not introduce significant structural damage.

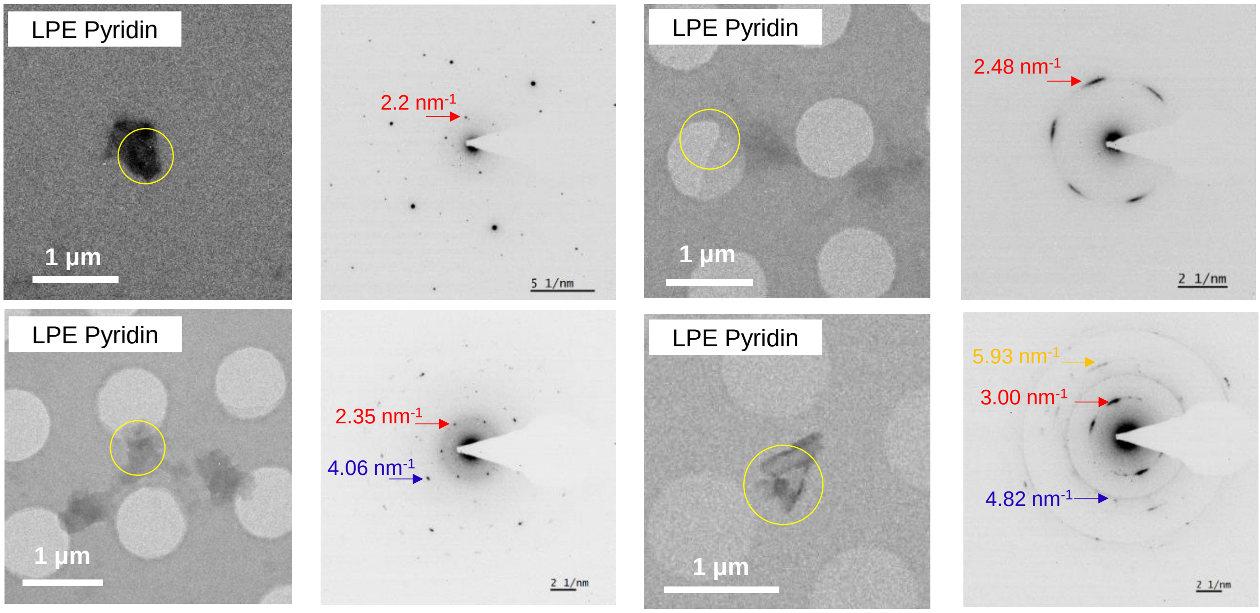

Figure S15: Bright-field TEM images and corresponding SAED patterns of the pyrylium-based 2D polymer **2** after exfoliation in aqueous sodium cholate. The 0.4-1k *g* fraction was investigated with an electron dose of 0.1 $e^-/Å^2$. In contrast to the pyrylium-based polymer **1** (figure S3), the appearance of higher order reflections in the SAED patterns demonstrates higher crystallinity of the exfoliated 2D polymer **2**.

IV.3 IR-Reflection-Absorbance Spectroscopy (IRRAS)

For both Raman and IRRAS, it was required to deposit the nanosheets as thin films on a reflective substrate such as Au-coated Si/SiO_2. Various deposition attempts by drop-casting and spin-coating, did not result in sufficiently homogeneous coverage for the measurements. The best result was obtained by dip-coating the Au-coated substrate into dilute dispersions (concentration ~0.01 gL^{-1}) of the nanosheets after removal of excess surfactant. This was achieved by redispersing the sediments after liquid cascade centrifugation with H_2O without addition of sodium cholate. A fraction containing large (0.4-1k *g*) and small (70-270k *g*) nanosheets was analyzed. The AFM images after deposition (Figure S16) confirm a homogeneous coverage with nanosheets.

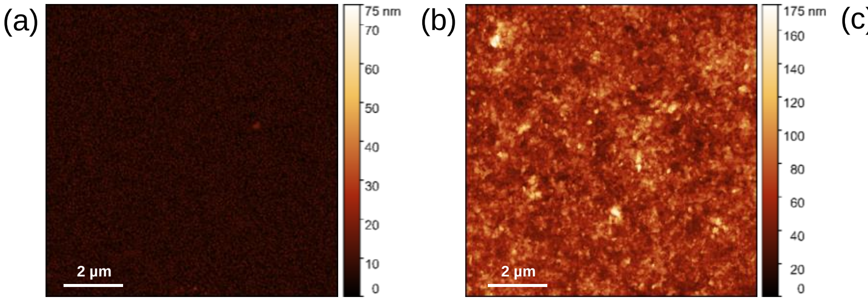

Figure S16: AFM images of nanosheets deposited in Au-coated Si/SiO_2 by dip-coating. a) AFM image of a wafer prior to deposition. Grain typical for sputtered Au are observed. b) Wafer after deposition of large nanosheets (0.4-1k *g*). c) Wafer after deposition of small nanosheets (70-270k *g*)

IRRAS measurements were performed on two different spots of the wafers. No significant spot to spot variations were observed as shown by the as-recorded (Figure S17A) and baseline subtracted (Figure S17B) spectra. Due to the complexity of the structure the modes could not be assigned. Unfortunately, IRRAS only probes a subset of the vibrations, i.e. those with a dipole moment

perpendicular to the surface. Therefore, a direct comparison to the ATR FTIR data of the bulk crystal is not possible.

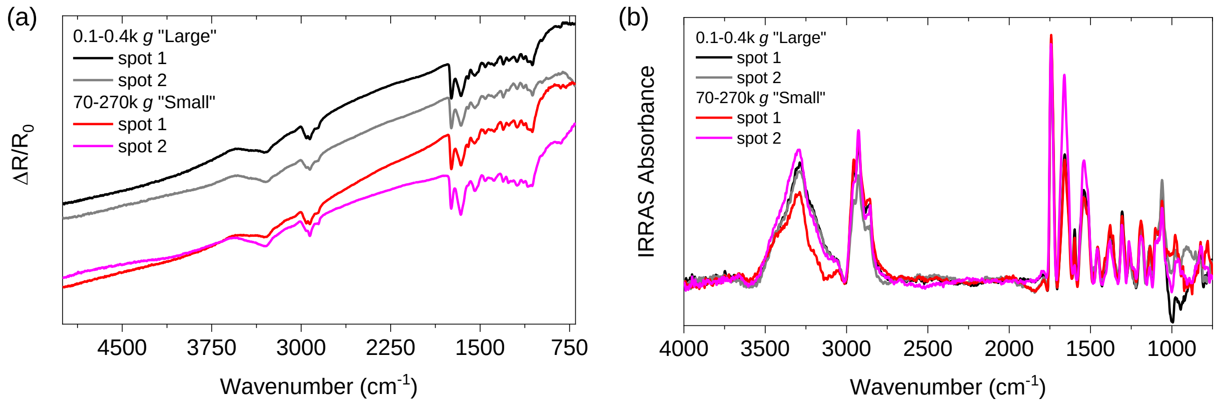

Figure S17: Infrared-Reflection-Absorption spectra (IRRAS) of two fractions of liquid-exfoliated nanosheets enriched in larger (0.1-0.4k g) and smaller (70-270k g) nanosheets after deposition on Au-coated Si/SiO_2. Two different spots on the substrate were analyzed. a) As-recorded spectra (after subtraction of the substrate. b) Normalized IRRAS absorbance spectra after baseline subtraction. No significant spot to spot variations are observed. The spectra of large and small nanosheets are identical except for some variations in relative peak intensities.

IV.4 AFM analysis nanosheet folding

Additional nanosheet images are provided in figure S18. The respective layer numbers are indicated in the figure. As clearly seen from the images of monolayers in the top row, the edges of the monolayers are not always as sharp as edges of few-layers. This suggests that sonication-induced scission occurred to some extent on exfoliation. Furthermore, thin nanosheets (1-4 layers) often appear folded when they are larger than 150 – 200 nm.

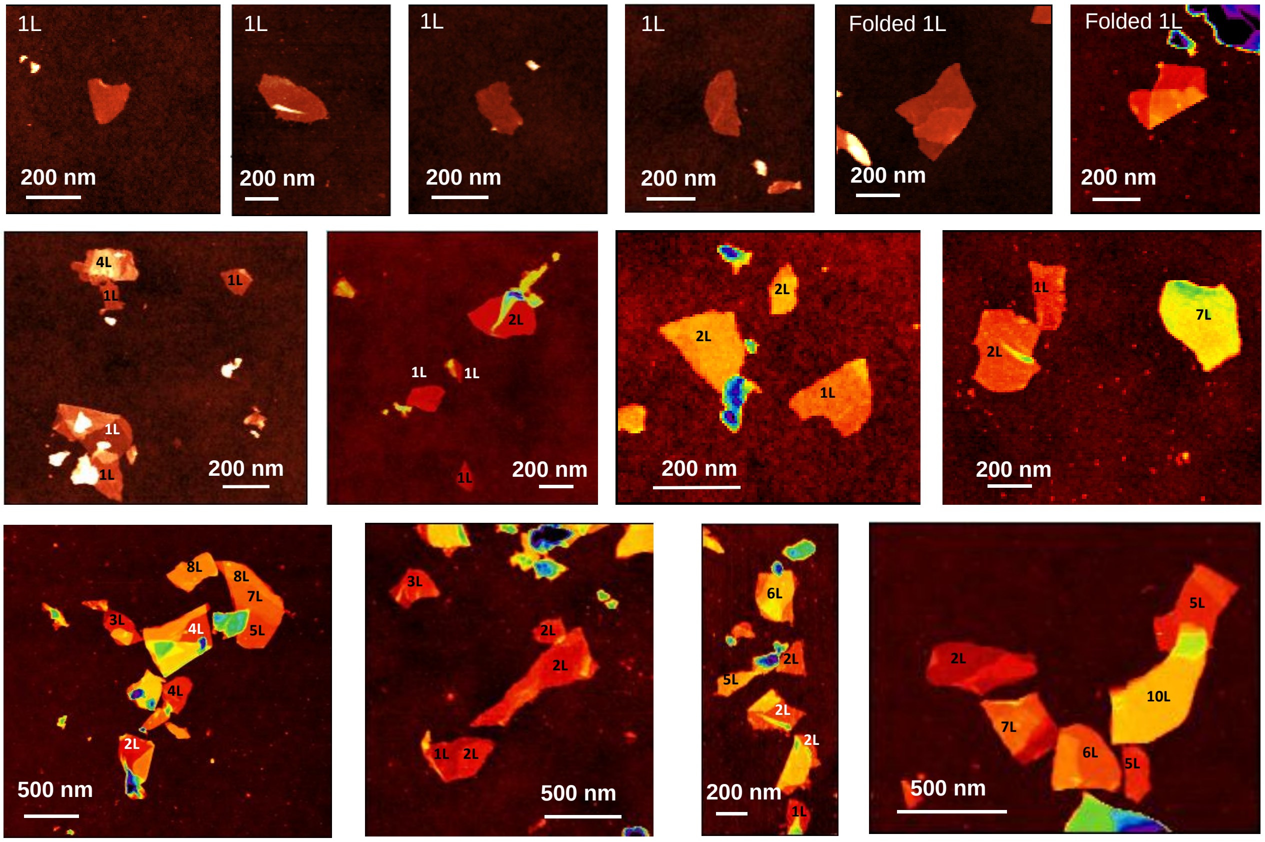

Figure S18: Additional AFM images with layer numbers indicated. Folded nanosheets are labelled by white numbers.

The folding is analyzed in more detail in figure S19. In figure S19a, the fraction of folded nanosheets is plotted as function of layer number. Nanosheets from all different dispersions were combined. In the case of monolayers, folding is observed in ~25% of the nanosheets. 2-4-layered nanosheets also appear folded frequently, while hardly any folding is observed for nanosheets > 5 layers. In general, the fraction of folded nanosheets decreases exponentially with increasing layer number. It should be noted that folding is expected to be more likely as nanosheets become laterally larger. This is confirmed by figure S19b which plots the mean nanosheet length as function of layer number for folded and non-folded nanosheets. In all cases, folded nanosheets are laterally larger than non-folded ones, as further illustrated by figure S19c which shows the lateral size of nanosheets with different layer number on a scatter plot. Again, folded nanosheets tend to be laterally larger than the group of non-folded nanosheets of the same layer number.

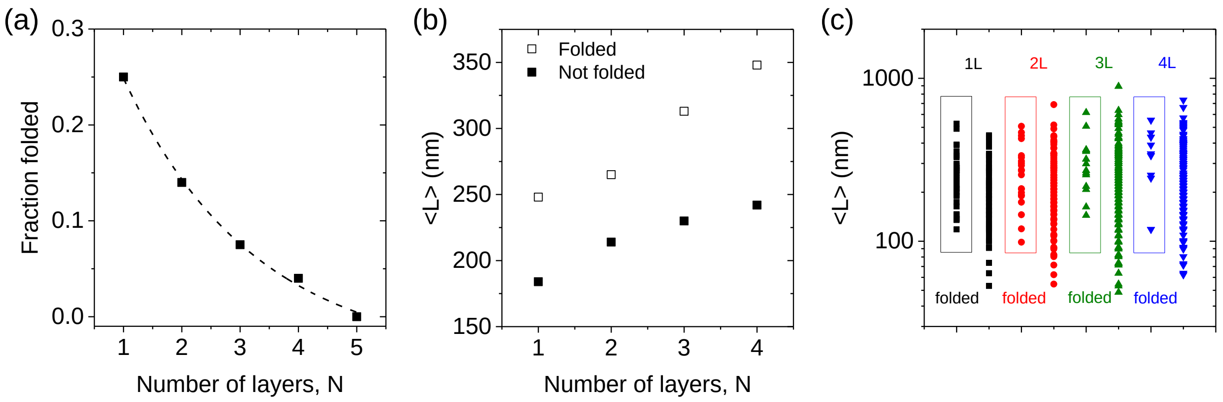

Figure S19: Analysis of the folding of nanosheets. **a)** Fraction of folded nanosheets as function of layer number. The fraction of folded nanosheets becomes negligible > 4 layers. **b)** Plot of the mean length of folded and non-folded nanosheets, respectively as function of layer number. Folded nanosheets are larger in average suggesting that folding become more prominent when nanosheets are larger. **c)** Plot of the mean nanosheet length as function of layer number in a scatter plot. Each data point represents one nanosheets. The data is grouped into folded and non-folded nanosheets. The group of folded nanosheets is indicated by the rectangles.

IV.5 Size selection procedures

IV.5.1 Standard cascade

The details of the standard centrifugation cascade are summarized in figure S20. After the first centrifugation step at low *RCF* (400 *g*), the sediment containing predominantly bulk material is discarded. The first samples that is collected was originally the supernatant from the 400 *g* centrifugation run which was then redispersed from the sediment after centrifugation at 1 000 *g*. The sample is thus denoted as 0.4-1k *g* indicating the lower and upper boundary of the centrifugation cascade and contains the larger/thickest nanosheets. Smaller/thinner nanosheets are collected as sediments as the dispersion progresses through the cascade. In total, six size-selected samples are obtained.

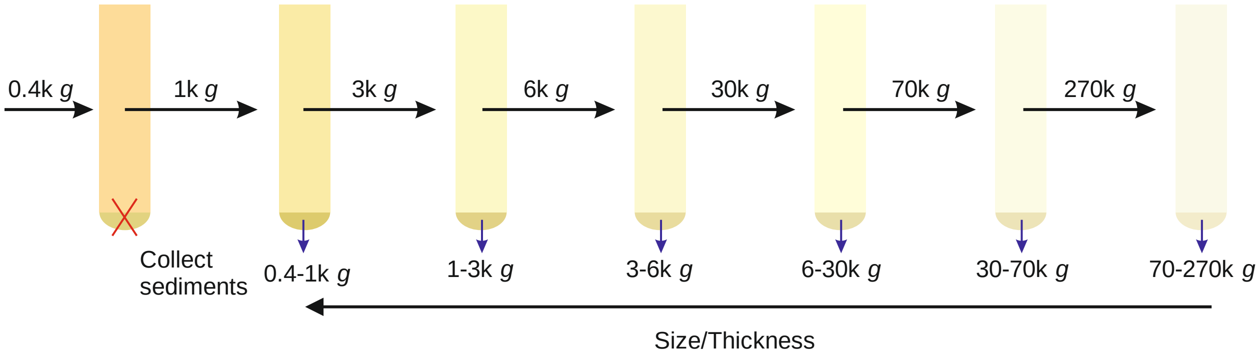

Figure S20: Schematic of the size selection by the standard cascade. After each step of the centrifugation, the sediments were collected in aqueous sodium cholate (0.1 g L^{-1}) at reduced volume (~1.5 mL from initially 25 mL). In this way, five dispersions were obtained containing nanosheets with different lateral size and thickness distributions.

IV.5.2 Secondary cascade

It has previously been shown that liquid cascade centrifugation cannot only be used to produce nanosheet dispersions with broad variations in nanosheet length and thickness, but also to enrich dispersions in monolayers without drastically sacrificing nanosheet length.[2] To this end, secondary cascades were previously designed for example involving overnight centrifugation runs at centrifugal accelerations below the initial lower trapping boundary.[2] To test whether this concept also works for the 2D polymer, the sample 6-30k *g* was subjected to a secondary cascade as illustrated in figure S21. This sample was chosen as it contains a reasonable monolayer content (~ 7% by number) in combination with <L> larger than 200 nm. After an overnight centrifugation at 1 000 *g*, both supernatant and sediment were subjected to AFM to confirm the result. The supernatant was then centrifuged at 7 000 *g* for 7h and then analyzed. In addition, a dispersion with an even higher ML content, namely 30-70k *g* (24% by number) was subjected to overnight centrifugation at 6k *g*.

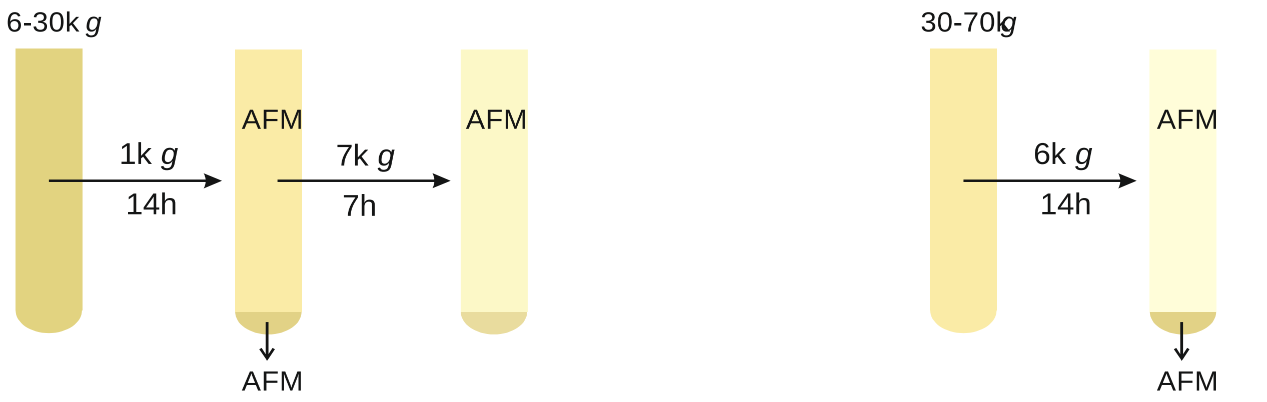

Figure S21: Scheme of the secondary centrifugations to demonstrate monolayer enrichment for the samples 6-30k *g* and 30-70k *g*.

IV.6 Size selection characterization

IV.6.1 AFM

To confirm the exfoliation and size selection and gain insights into the structure of the constituents in the dispersion, the size-selected samples were deposited on Si/SiO_2 wafers and subjected to atomic force microscopy (AFM). Typical wide-view images of the dispersions produced from the standard cascade are displayed in figure S22 and show 2D nanosheets with lateral sizes ranging from ~100 nm to a few µm. This clearly confirms the success of the exfoliation. As expected, larger/thicker nanosheets are enriched in the samples collected at the beginning of the cascade, while/smaller thinner nanosheets dominate in the fractions collected at higher centrifugal accelerations.

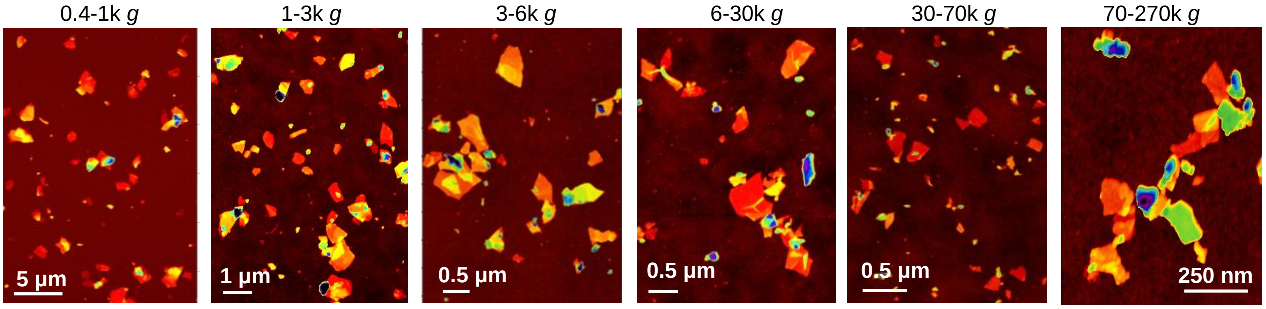

Figure S22: Representative wide-view atomic force micrographs of the size-selected dispersions produced in the standard cascade.

AFM was then used to statistically analyze the nanosheet dimensions. In each dispersion 200-300 individual nanosheets were measured and their length (longest dimension), width (perpendicular to length) and thickness recorded. In the case of incompletely exfoliated nanosheets, the thickness was averaged across the nanosheet. It should be noted that in addition to the nanosheets, we sometimes find non-2D deposits (figure S23) which are typically thicker than the

nanosheets. This is particularly the case in the samples isolated at higher centrifugal acceleration. The nature of these impurities is currently unknown. These were excluded from the statistical analysis.

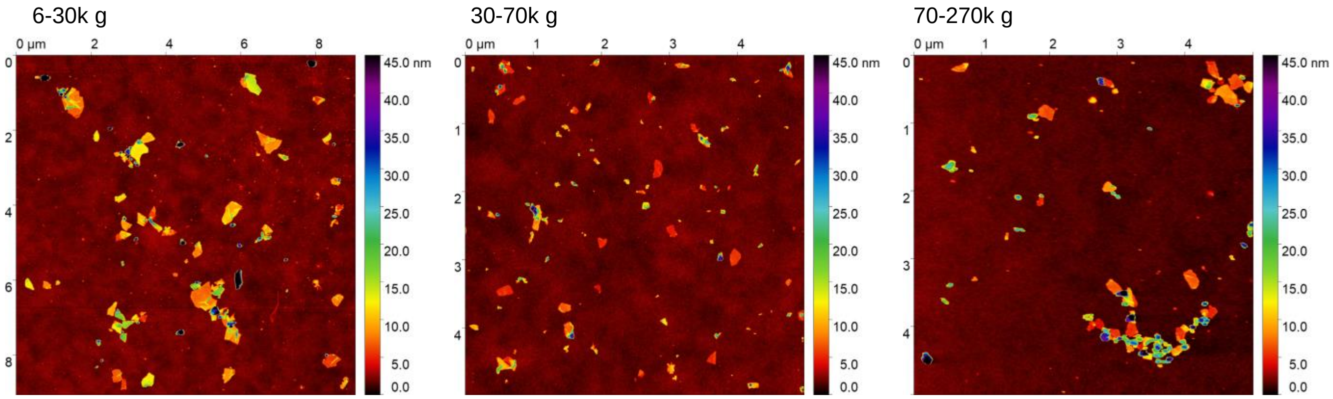

Figure S23: Wide view AFM images showing non-2D deposits in addition to the nanosheets.

To correct the lateral size for cantilever and pixilation effects, we applied previously established length corrections[15] and nanosheet thickness was converted to layer number via step height analysis as described in the main manuscript. After such length and thickness corrections, layer number (N) distribution histograms and length (L) distribution histograms of the size-selected samples are obtained. These are displayed in figure S24. All histograms are lognormal in shape with long tails especially in the thickness histograms (top row). Importantly, the samples 6-30k *g* and 30-70k *g* and 70-270k *g* contain a significant portion of monolayers.

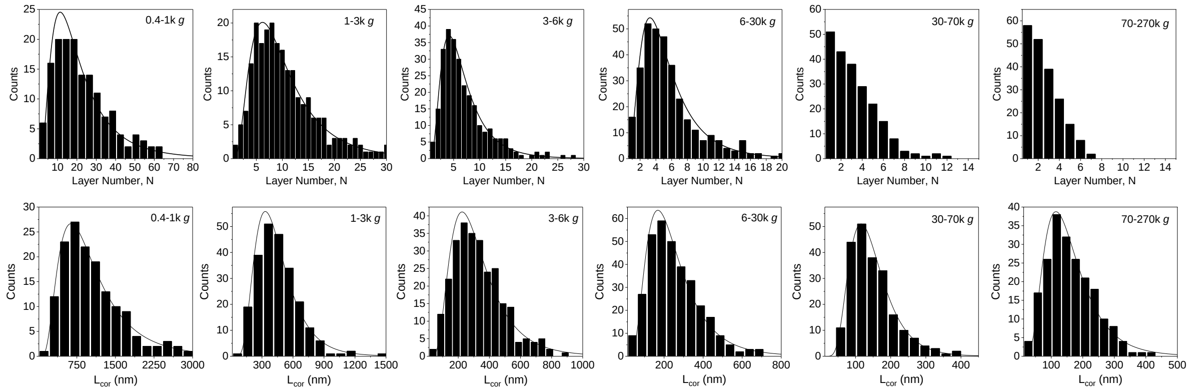

Figure S24: Layer number (top) and length (bottom) distribution histograms of the dispersions isolated in the standard cascade.

Representative images and distribution histograms of the samples produced from the secondary cascade are displayed in figure S25 in comparison to the histograms of the respective samples prior to overnight centrifugation. The most significant change is a reduction in the long tail of the distribution and a narrowing of the thickness distribution which leads to the observed increase in monolayer content.

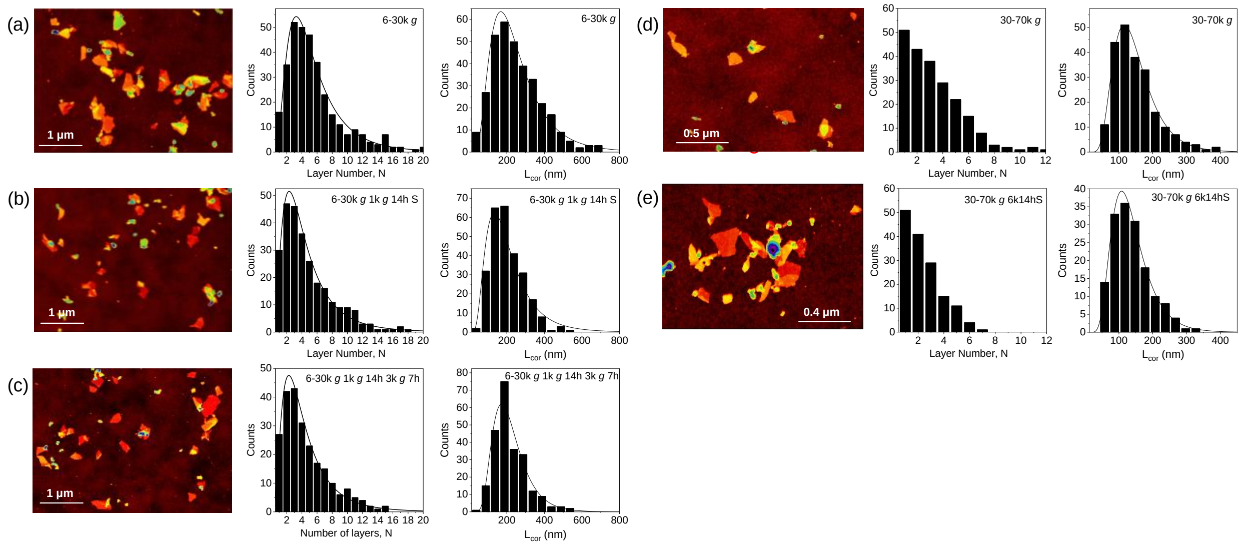

Figure S25: AFM characterization of the secondary cascades starting from the 6-30k g (left) and 30-70k g (right) dispersions, respectively.

IV.6.2 Scaling of nanosheet dimensions

In addition to the central plots shown and discussed in the main manuscript, the size selection and quantification can be analyzed in more detail. As implied in the main manuscript, length and thickness are simultaneous varied on size selection using the standard cascade. This means that <L> and <N> are linked and scale with each other by a power law as shown by the plot in figure S26a. It should be noted that this is not necessarily a result of the centrifugation procedure, as a similar scaling is observed when plotting the length as function of thickness for the individual nanosheets as illustrated by the scatter plot in figure S26b. Such a behavior has been observed before for other materials[16] and is likely related to the energetics of the exfoliation, as it requires more net energy to overcome the interaction between laterally larger nanosheets over their smaller counterparts. As a result, larger nanosheets tend to be thicker and thinner nanosheets tend to be smaller. As a result, it is extremely challenging to enrich thin, but large nanosheets in a dispersion.

In the main manuscript, we use the arithmetic mean to express the thickness of the nanosheets in a given dispersion. However, similar to the difference between number average molecular weight and weight average molecular weight in polymer physics, the mean thickness can also be expressed by the volume fraction weighted mean, $\langle N_{Vf} \rangle$ which can be calculated from the AFM statistics once length, width and thickness are known using $\langle N \rangle_{Vf} = \sum N^2 LW / \sum NLW$, where the summations are over all nanosheets. This is plotted as function of central *RCF* in figure S26c and also scales as a power law just like the arithmetic mean (but with a different exponent). It might seem that the volume fraction weighted mean is a more accurate description of the system. However, as previously found for liquid-exfoliated WS_2,[15] the arithmetic and volume fraction weighted mean thickness scale linearly with each other as shown in figure S26d so that both values

are an adequate description of the constituents in the dispersion. In the plot, data from the secondary cascades is included (red data points) and falls on the same curve with data from the standard cascade. We note that this plot is of great value, as it can be used to validate the accuracy of the AFM statistics. This is because an insufficient number of data points in the statistic leads to non-uniform distributions and causes a deviation when plotting the arithmetic mean as function of volume fraction weighted mean.

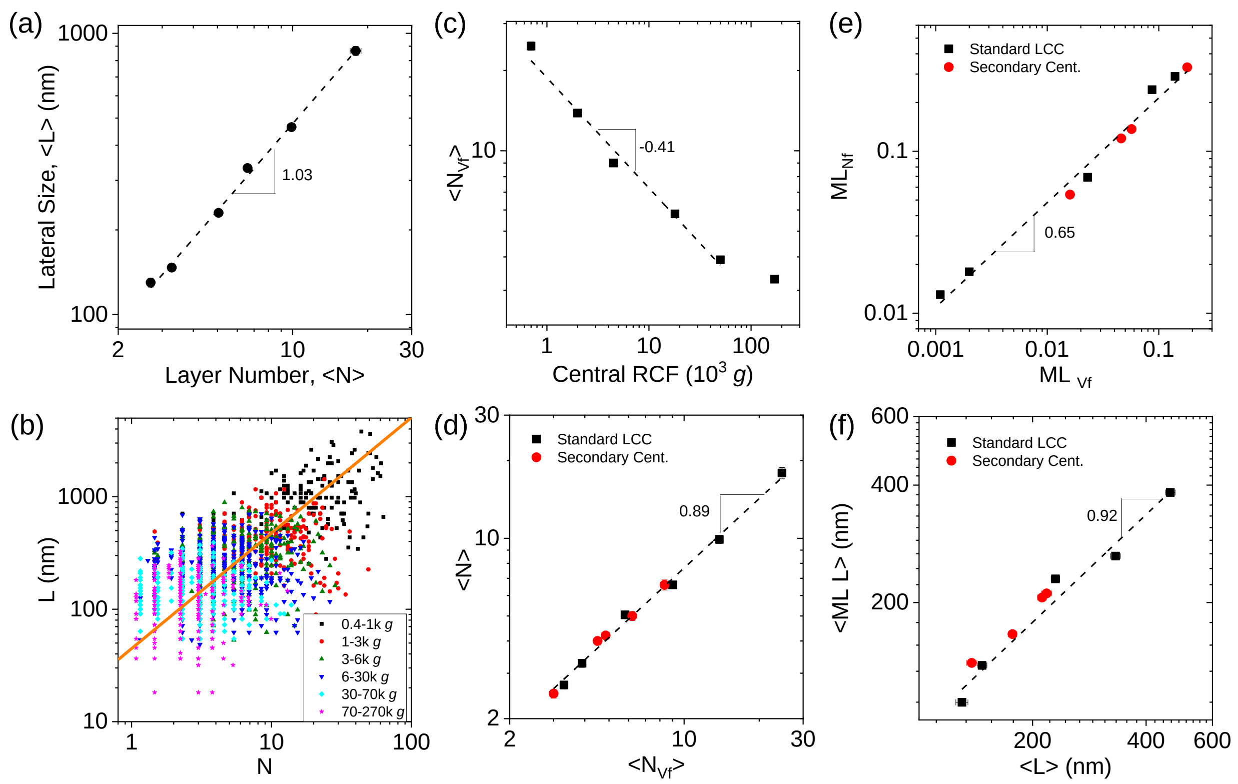

Figure S26: Scaling of nanosheet dimensions. **a)** Plot of mean nanosheet length as function of mean layer number. **b)** Scatter plot of length as a function of layer number. Each data point represent an individual nanosheet measured with AFM. The fractions from the standard cascade are color coded. The orange line represents the scaling obtained from the mean values of the fractions. **c)** Plot of volume fraction weighted mean layer number as function of the centrifugation midpoint in the cascade. **d)** Relationship between arithmetic and volume fraction weighted mean thickness. **e)** Plot of monolayer number fraction as function of monolayer volume weighted fraction. **f)** Plot of mean monolayer length versus mean length over all nanosheets. In **(d-f)**, data from secondary cascades is included (red data points).

Similarly, the monolayer content can be expressed as number fraction (ML_{Nf}) or volume weighted fraction (ML_{Vf}). In analogy to <N> and <N_{Vf}>, we find a linear scaling (Fig. S26E) with data from the standard and secondary cascade falling on the same curve. The maximum volume weighted monolayer fraction that was achieved is ~0.18 (see table 1 in main manuscript). Another interesting aspect to consider is the lateral size of the monolayers. We find the monolayer length, <ML L> to scale linearly with the mean lateral size over all nanosheets with a slope close to 1 (Fig. S26F). This is an important aspect for further sample optimization, as the mean lateral size is much easier to measure than the lateral size of monolayers (which requires more extensive statistics) in particular when taking into account readily accessible spectroscopic metrics to determine <L> (see below).

As mentioned above, it is very difficult to decouple the intrinsic relationship between length and thickness. Secondary cascade have shown some promise to achieve that. The plot of length *versus* layer number (figure S27a) confirms that the secondary cascade (red data points) successfully produced dispersions with a L-N relationship significantly deviating from the samples produced in the standard centrifugation cascade (black data points). Importantly, this allowed us to make dispersions with a similar length of 215-240 nm, but $\langle N \rangle_{Vf}$ varying from 4-7 or <L> between 140-175 nm, but <N> varying from 2.5-5. This will be important to test the size-dependent optical properties (see below). Due to the link between <L> and monolayer length, the plot in figure S26b can be also used to illustrate that secondary cascades have the potential to produce fractions enriched in laterally larger nanosheets compared to standard cascades.

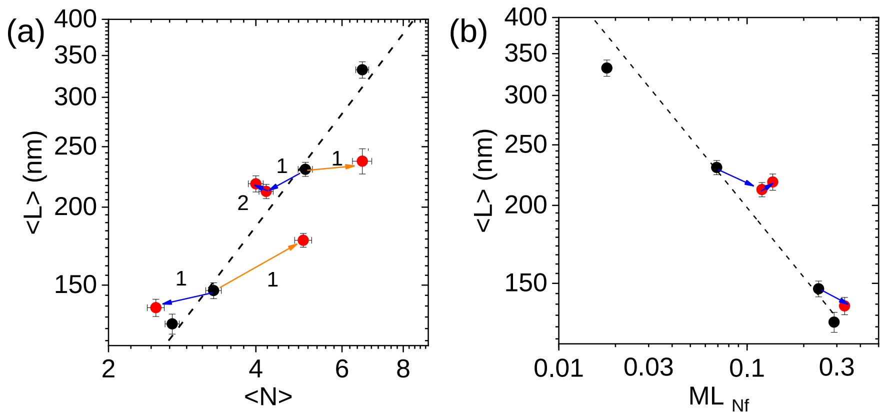

Figure S27: Scaling of nanosheet dimensions after the secondary centrifugation cascade. **a)** Plot of mean layer number as function of mean thickness. Data from the secondary cascade is shown in red clearly demonstrating that <L> and <N> can be decoupled. Blue arrows indicate supernatant samples, orange arrows sediments. The number indicates the centrifugation step (figure S21). **b)** Plot of mean layer number as function of monolayer number fraction. A decoupling can also be observed to some extent.

IV.7 Graphene reference experiment

To probably benchmark the exfoliation efficiency (in terms of produced nanosheet sizes and yield), we exfoliated and size selected graphene in a comparable way. Graphene dispersions were prepared by probe sonicating (VibraCell CVX, 750W) powder (Sigma Aldrich flakes 332461-2.5 kg) at a concentration of 20 g L-1 dispersed in a 6 g L^{-1} aqueous solution of sodium cholate (Sigma Aldrich BioXtra, ≥99%) for 1 hr at 60% amplitude. The dispersion was then centrifuged in a Hettich Mikro 220R centrifuge equipped with a fixed-angle rotor 1016 at 2260 *g* for 2 h. The supernatant was removed and the sediment was redispersed in fresh surfactant solution (c_{SC} = 2 g L^{-1}) and subsequently sonicated for 5 h at 60% amplitude with a pulse of 6 on and 2 off. The resultant stock dispersion was centrifuged at 27 *g* for 2 h, sediment discarded and the supernatant subjected to size selection. For the size selection of nanosheets, we used a centrifugation cascade increasing the speed and moving the supernatant on to the next stage each time. The sediment after each centrifugation was collected and redispersed in fresh surfactant solution. The speeds used were 0.1k *g*, 0.4k *g*, 1k *g*, 5k *g*, 10k *g*, 30k *g*. For centrifugation < 3k *g*, a Hettich Mikro 220R centrifuge equipped with a fixed-angle rotor 1016 (50 mL vials filled with 20 mL each) was used. For centrifugation > 3k *g*, a Hettich Mikro 220R centrifuge equipped with a fixed-angle rotor 1195-A (1.5 mL vials). All centrifugation was performed for 2 h at 15°C. Samples subjected to AFM and extinction spectroscopy in analogy to the 2D-polymer. To calculate the concentration (and thus yield), we used the size-independent extinction coefficient at 750 nm which was previously determined as 5450 L g^{-1} m^{-1}.[16d]

The key data for the reference experiment is summarized in figure S28 and table S3. Similar to the 2D polymer, we find <L> and <N> to decrease with the central centrifugal acceleration as a

power law (Fig. S28A-B) albeit with a different exponent. It should be stressed that smaller and thinner nanosheets are accessible at lower centrifugal accelerations due to the higher density of graphite/graphene compared to the porous 2D polymer.

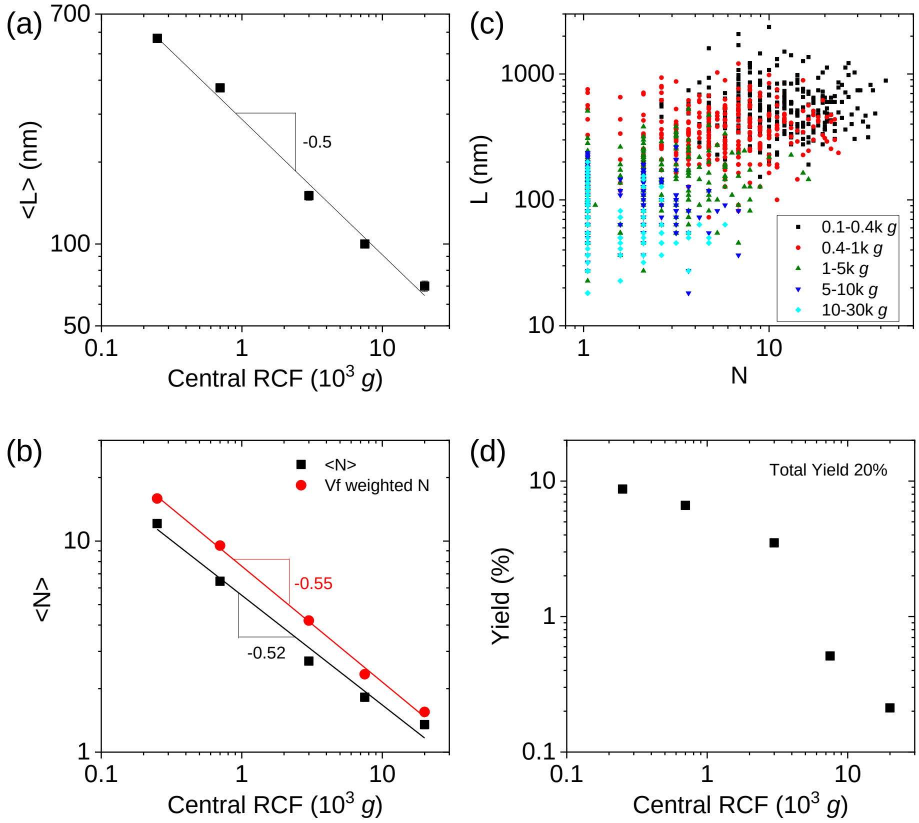

Figure S28: Key data of the exfoliation and size selection of graphite performed as reference experiment. **a)** Plot of mean nanosheet length as function of *RCF* and **b)** Nanosheet layer number as function of *RCF*. Both arithmetic and volume fraction weighted mean are displayed. **c)** Plot of length as function of layer number as a scatter plot where each data point is a nanosheet which size/thickness was measured by AFM. **d)** Plot of yield as a function of *RCF*.

We also find a similar relation between lateral size and nanosheet thickness on the scatter plot (Fig. S28C) illustrating a similar linkage between length and thickness. This results in the data of <L> versus <N> in the fractions to fall on the same line (see main manuscript). The yield of exfoliated graphite (i.e. mono and few-layer graphene) is plotted as function of central RCF in

figure S28d. It shows a similar decrease in nanosheet yield with increasing centrifugal acceleration as the 2D polymer. Importantly, we stress that the overall yield of few-layered nanosheets is 20% which is identical to the overall yield obtained for the 2D polymer.

Table S3: Tabulated values of graphene mean thickness <N>, mean length <L>, monolayer number fraction ML Nf, monolayer volume fraction ML Vf, total nanosheet yield, yield of monolayers, number of nanosheets and number of monolayer in each fraction collected.

<N>	12.1	6.5	2.7	1.8	1.4
<L>	570 nm	375 nm	150 nm	100 nm	70 nm
ML Nf	0	0.034	0.33	0.54	0.68
ML Vf	0	0.0046	0.078	0.28	0.56
Yield (%)	8.7	6.6	3.5	0.51	0.21
ML Yield (%)	0	0.03	0.27	0.14	0.11
# of all nanosheets	1.7 E12	6.0 E12	4.6 E13	2.6 E13	3 E13
# of ML	0	2 E11	1.4 E13	1.5 E13	2.1 E13

IV.8 Size dependent optical properties – quantitative length and thickness metrics

A number of inorganic 2D materials[2, 16a, 16c, 17] and graphene[16d] all exhibit size-dependent optical properties. In particular, extinction and absorbance spectra show well defined changes with nanosheet lateral size and layer number due to edge and confinement effects, as well as contributions from non-resonant light scattering.[16a] To test whether this is also the case for the 2D polymer, the size-selected dispersions were subjected to optical measurements. We used both extinction and absorbance spectroscopy. [16a, 18] It should be noted that the extinction (Ext) is a

combination of both the absorption (Abs) and scattering (Sca) where Ext(λ) = Abs(λ) + Sca(λ).[19] This means, an inherent variation across the dispersions can be anticipated in the extinction spectra due to non-negligible contributions from light scattering which is expected to be size dependent. This also implies that extinction and absorbance coefficients which can be used to determine the nanosheet concentration are not only wavelength, but also size-dependent.

We therefore first analyse extinction and absorbance coefficient spectra of dispersions where the nanosheet concentration was determined gravimetrically after filtration and weighing. Extinction coefficient spectra are also shown in the main manuscript, but compared to absorbance coefficient spectra in figure S29. Absorbance spectra were acquired by measuring the spectra with the dispersion placed in the centre of an integrating sphere, where scattered light is collected by the reflective coating.

As expected, systematic variations in the spectral profile are observed in the case of the extinction coefficient spectra (figure S29a). For example, above 400 nm the extinction follows a power law decay. This background is more pronounced for larger/thicker nanosheets strongly suggesting that this is due to light scattering. This is confirmed by the absorbance coefficient spectra (figure S29c) which show no signal in this spectral region. Nonetheless, these absorbance spectra also exhibit sample dependent changes which we attribute to edge and confinement effects in addition to the size dependent changes in the extinction coefficient spectra which are due to light scattering. According to these absorbance coefficient spectra, the exfoliated 2D polymer can be classified as wide bandgap semiconductor with an optical gap of ~4.4 eV with a slight dependence of the band edge on nanosheet length/thickness.

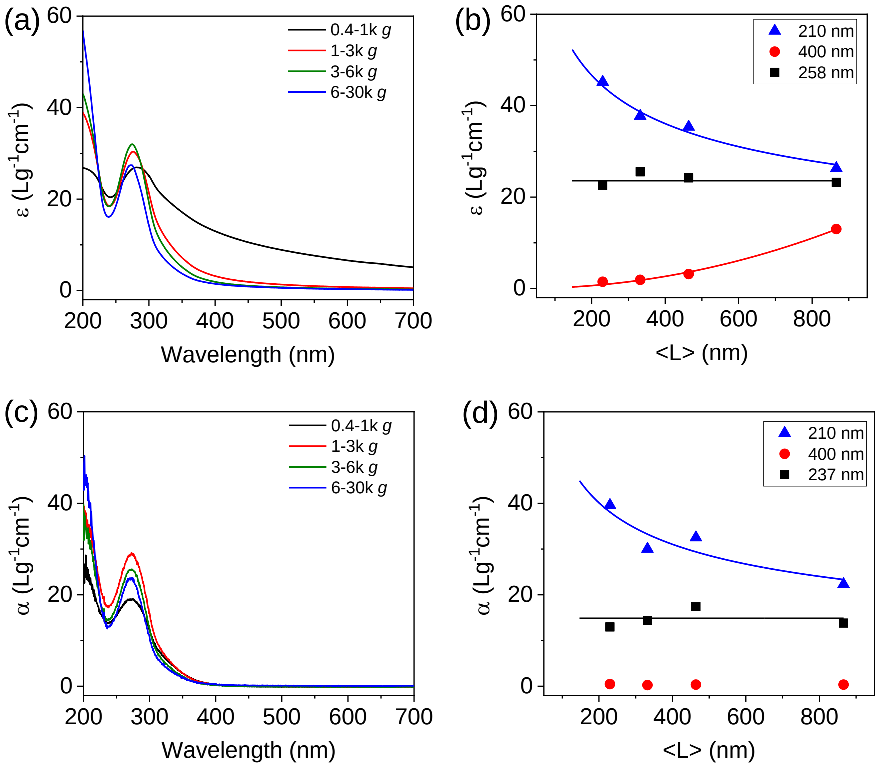

Figure S29: Analysis of extinction and absorbance coefficient spectra. **a)** Extinction coefficient spectra as function of wavelength for different dispersions where the concentration of the nanosheets was determined gravimetrically to convert extinction to extinction coefficient via the Lambert Beer law. **b)** Plot of extinction coefficients at three wavelengths as function of nanosheet mean length <L>. **c)** Related absorbance coefficient spectra after measurement in an integrating sphere. **d)** Plots of absorbance coefficient as function of <L>.

To analyze these coefficient spectra in more detail, we plot the extinction/absorbance coefficient as function of nanosheet length for three wavelength each in figure S29b,d, respectively. As outlined in the main manuscript, the extinction coefficient is size-independent at 258 nm so that this wavelength can be used for a determination of the nanosheet concentration. In the nonresonant regime, i.e. at 400 nm, it increases exponentially with nanosheet size. The absorbance coefficient (Fig. S29d) is in turn zero so that the extinction coefficient at this wavelength is essentially a

measure of the scattering strength which scales in a well-defined way with nanosheet dimensions. This is in contrast to wavelengths in the resonant regime, for example 210 nm. In this wavelength regime, both extinction and absorbance coefficient decrease exponentially with nanosheet size which we attribute to varying contributions from nanosheet edges and basal plane to the absorbance (see below). Also in absorbance coefficient spectra we find a point where the coefficient is invariant with nanosheet size (Fig. S29D). In this case, this is observed at the local minimum at 237 nm, i.e. slightly blue shifted compared to the wavelength of the size-independent extinction coefficient. This is because scattering spectra in the resonant regime follow absorbance spectra in shape, albeit red-shifted due the dependence of the resonant scattering with refractive index of the dispersed material.[20]

Importantly, since the absorbance and extinction coefficient spectra are of comparable in shape in the resonant regime, extinction spectra rather than absorbance spectra can be analyzed to extract further information. For convenience, we present spectra normalized to 237 nm in figure S30a also including spectra where the coefficient spectra were not accessible due to the low mass of the material in the fractions produced at high centrifugal acceleration. All but the smallest/thinnest fraction show well-defined change with nanosheet/size thickness.

To analyze peak positions in addition to overall shape, it has been proven useful to plot the second derivative of peaks to reveal fine-structure that is not otherwise accessible.[2] The second derivative in the region of the resonant peak (Fig S30B) reveals a hidden fine structure resembling vibrational structure of molecules and will be investigated in more detail below.

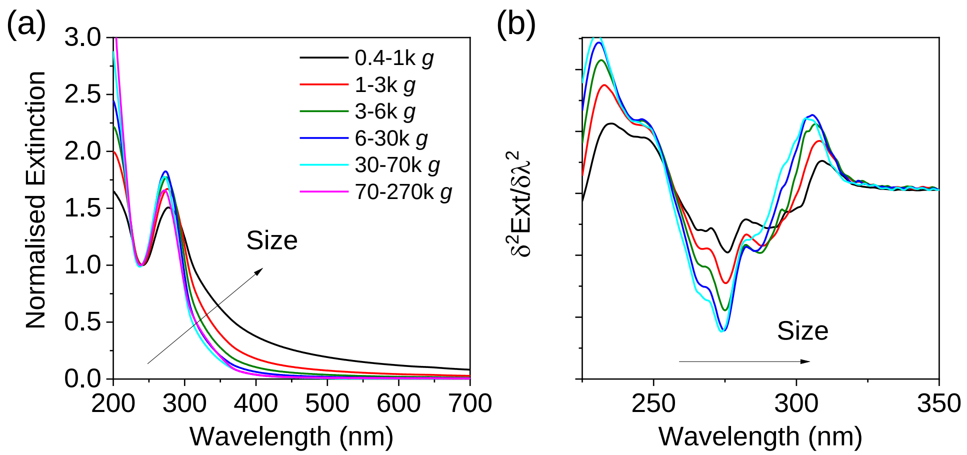

Figure S30: Extinction spectra over a range of samples. **a)** Extinction spectra normalized to the local minimum for a range of 2D polymer samples as indicated in the figure legend. **b)** Second derivative spectra in the spectra region of the main peak.

The size-dependent optical response described above gives scope for quantitative metrics to relate the nanosheet lateral size and/or thickness to the spectral profile. This will be extremely important to optimize both exfoliation and size selection in the future, as tedious and time consuming AFM statistics can be minimized. It has previously been suggested that edge effects are manifested by different absorbance coefficients at edge and central region, respectively.[16a] As a result, peak intensity ratios in the resonant regime are expected to scale with the nanosheet lateral size in a well-defined way according to equation 1:

$$\frac{Ext_{\lambda 1}}{Ext_{\lambda 2}} = \frac{\varepsilon_c(\lambda 1)L + 2x(k+1)\Delta\varepsilon(\lambda 1)}{\varepsilon_c(\lambda 2)L + 2x(k+1)\Delta\varepsilon(\lambda 2)} \qquad \text{Equ 1}$$

where ε_c is the extinction coefficient associated with the nanosheet basal plane, $\Delta\varepsilon = \varepsilon_E - \varepsilon_c$ where ε_E is the edge region extinction coefficient, and L, x and k are the nanosheet length, thickness of the edge region and length-width aspect ratio, respectively.

In figure S31a, an extinction peak intensity ratio in the resonant regime, i.e. Ext_{270nm}/Ext_{350nm} is plotted as a function of mean nanosheet length. Data from the standard cascade (black data

points) and the secondary cascade (red data points) is included and falls on the same curve. Unfortunately, data from the monolayer-rich dispersions produced after ultracentrifugation at 270k *g* could not be used for the analysis due to the presence of a shoulder at 330-350 nm likely stemming from impurities in the sample. The dashed line is a fit to equation 1 and describes the data very well confirming that similar edge effects contribute to the size-dependent optical properties as previously observed for TMDs.[2, 16a] This allows us to generate a function to quantitatively relate the extinction peak intensity ratio Ext_{270nm}/Ext_{350nm} to the mean nanosheet length according to equation 2:

$$<L>=\frac{4992-243.9\frac{Ext270nm}{Ext350nm}}{\frac{Ext270nm}{Ext350nm}+3.27} \qquad \text{Equ 2}$$

Alternatively, a peak intensity ratio in the non-resonant regime and a wavelength in the resonant regime can be used to express the contribution from scattering to the extinction spectra. As an example, the ratio Ext_{500nm}/Ext_{240nm} is plotted as function of nanosheet length in figure S31b. Again, the data from the standard cascade and the secondary cascade, respectively, falls on the same curve. The dashed line is an empirical exponential fit which allows us to establish a second length metric according to equation 3:

$$<L>=1053\ ln\frac{\frac{Ext500nm}{Ext240nm}+0.187}{0.167} \qquad \text{Equ 3}$$

Note that this metric will only be accurate as long as the scattering contribution is not negligible, i.e. for nanosheets > 200 nm, as the ratio drops to almost 0 for nanosheets < 150 nm.

Perhaps of more interest than metrics for nanosheet length are metrics for nanosheet layer number. Spectroscopy essentially averages over the volume of the sample probed, so technically the volume fraction weighted mean thickness should be correlated to the peak position. However,

due to the scaling of the arithmetic mean and the volume fraction weighted mean described above, <N> can also be used.

In the case of TMDs[2, 16a] and GaS,[16c] we have empirically found that the peak energies of the excitonic transitions scale exponentially with layer number due to confinement and dielectric screening effects. To test this for the 2D polymer, we have chosen two peaks of the second derivative of the extinction spectra (compare figure S30b) and determined the peak positions. These were then converted to eV and plotted as function of layer number in figure S31c and S31d, respectively. In both cases, we see an exponential decrease with increasing layer number. Error bars in the peak positions were estimated from the 0.5 nm increments used to acquire the spectra which corresponds to ~0.007 eV in this spectral region. Importantly the data from the secondary cascade, where <N> and <L> were decoupled, fall on the same curve confirming that the peak positions are indeed related to nanosheet thickness rather than length. From the fit curves we obtain two equations which both relate the mean nanosheet thickness to peak positions from the second derivative of the extinction spectra according to equations 4 and 5, respectively.

$$< N >= 1 + 4.75 \ln \frac{0.0445}{Pos1 - 4.5} \qquad \text{Equ 4}$$

$$< N >= 1 + 14.9 \ln \frac{0.145}{Pos2 - 4.22} \qquad \text{Equ 5}$$

Since the monolayer content is related to the mean thickness of the nanosheets in a dispersion, this indirectly gives an estimate to monolayer content from optical spectra. We note that these metrics will not only be extremely powerful for further sample optimization, but are also interesting in its own right, as they suggest that 2D materials show a generic size-dependent behavior irrespective of their chemical composition.

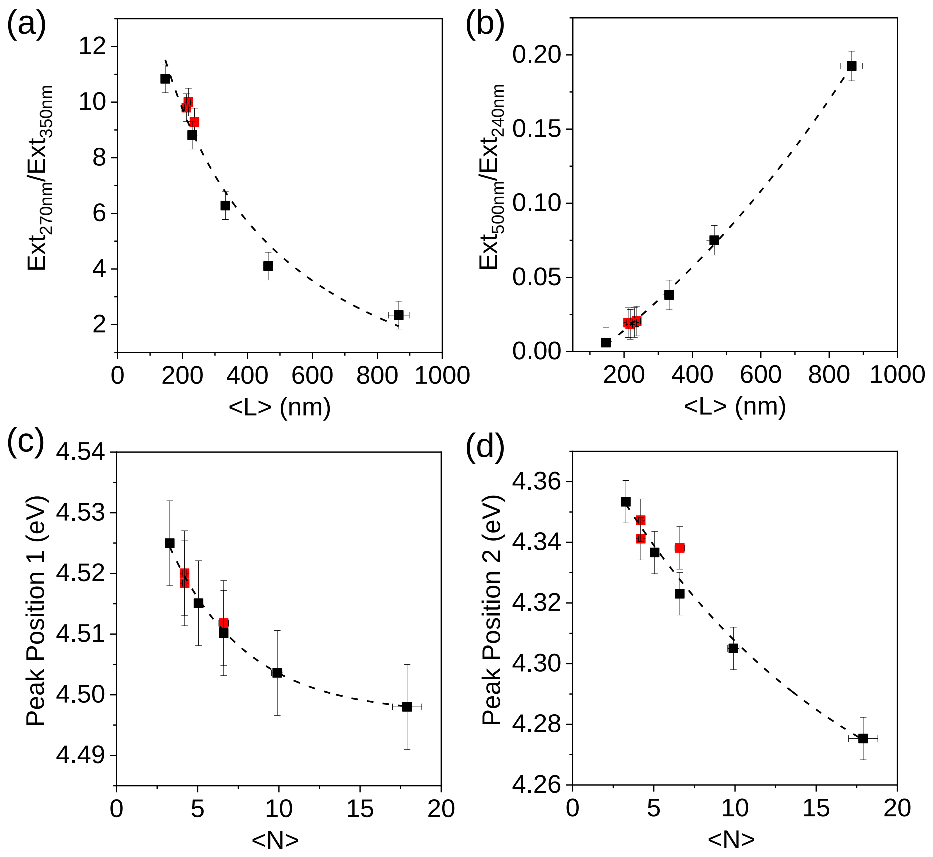

Figure S31: Spectroscopic metrics to determine nanosheet length and thickness. **a)** Plot of the extinction peak intensity ratio in the resonant regime at 270 nm to 350 nm (Ext_{270nm}/Ext_{350nm}) as function of <L>. The dashed line is a fit to equation 2. **b)** Plot of the extinction peak intensity ratio in the non-resonant regime at 500 nm to 240 nm (Ext_{500nm}/Ext_{240nm}) as function of <L>. The dashed line is an empirical exponential fit. **c-d)** Plots of extinction spectra peak positions as function of layer number. The dashed lines are empirical exponential fits. In all cases, the red data points represent the samples from the secondary cascade with similar length, but significant variations in thickness. These fall on the same plots with the samples from the standard cascade suggesting that length and thickness metrics are widely independent from each other.

IV.9 Sediment recycling

Since the overall yield of the exfoliation is only 20%, it is important to test whether the material that was removed as unexfoliated crystallites can be recycled. The sediment obtained after centrifugation at 400 *g* was therefore filtered (microfiltration, Al_2O_3 membranes), washed with water and dried. This powder was then subjected to tip sonication under identical conditions and the same cascade centrifugation performed. Nanosheet yield, lateral size and layer number was evaluated from the optical extinction spectra (Figure S32) using the spectroscopic metrics described above and plotted in figure S33 as function of central *RCF* together with the data from the initial exfoliation. For length and thickness of the recycled sediment samples, two numbers are given according to the two different metrics for length and thickness, respectively (see Equ. 2-4 above). The yield (Figure S33a) is comparable to the initial exfoliation, albeit a bit lower for fractions isolated at high *RCF*. The total yield of the second exfoliation is 16%, i.e. relatively close to the initial 20%. Both lateral sizes and thicknesses from both exfoliation runs match well with the exception that slightly larger nanosheets are obtained after the second exfoliation.

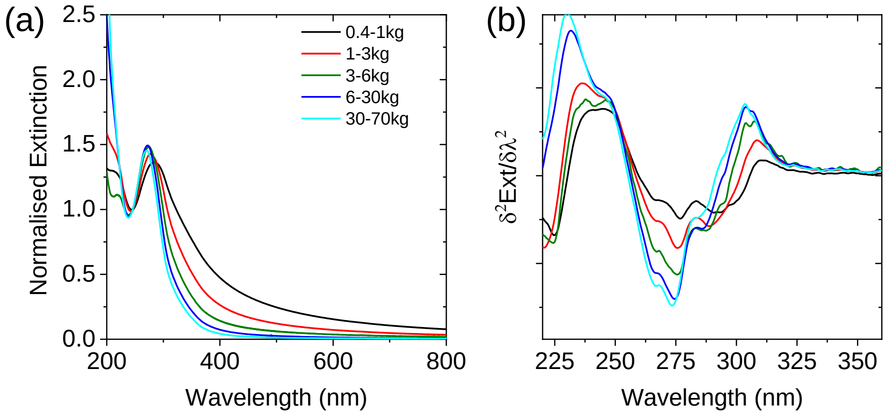

Figure S32: **a)** Normalized optical extinction spectra and **b)** second derivative of the exfoliation of **2** that was removed as unexfoliated material after the initial exfoliation.

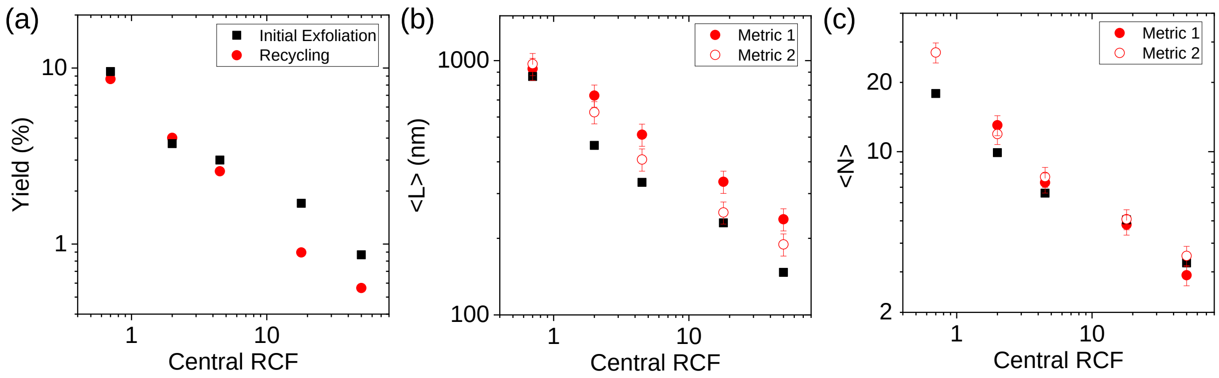

Figure S33: Comparison of **a)** exfoliation yield, **b)** mean nanosheet lateral size and **c)** mean nanosheet layer number as function of central *g* force of the initial exfoliation and the sediment recycling experiment. The data for the recycled sediment was obtained using the spectroscopic metrics described above.

IV.10 Calculation of the number of nanosheets

The calculation of the number of nanosheets can be performed with knowledge of the molar mass of **2** in the unit cell, M and the area of the unit cell, A_{UC} with M=2857.4 g L^{-1} and A_{UC} = 6.32 nm^2 in combination with the determined nanosheet mass, m and mean nanosheet area <LW>.

The total number of sheets, N, is given by

$$N = N_A \frac{m}{\frac{< LW >}{A_{UC}} * M}$$

With N_A being the Avogadro constant.

In analogy, the number of monolayers, N_{ML} in the fraction can be calculated as

$$N_{ML} = N_A \frac{m_{ML}}{\frac{< LW >_{ML}}{A_{UC}} * M}$$

Where <LW>$_{ML}$ is the area of the monolayered nanosheets and m_{ML} the mass of monolayer nanosheets which is related to the total mass via the monolayer volume fraction.

IV.11 Characterization of the fraction of unexfoliated 2

We subjected the largest/thickest fraction of nanosheets discarded as “unexfoliated” to TEM. As the thick fraction contains a higher number of layers in the nanosheets, we expected that the nanosheets could withstand a higher electron dose before structural disintegration, thus giving rise to clear diffraction patterns. However, we found that a substantial portion of the nanosheets were amorphous, showing no diffraction spots even under the electron dose of merely 0.1 $e^-/Å^2$. Results of the TEM analysis are shown in figure S34.

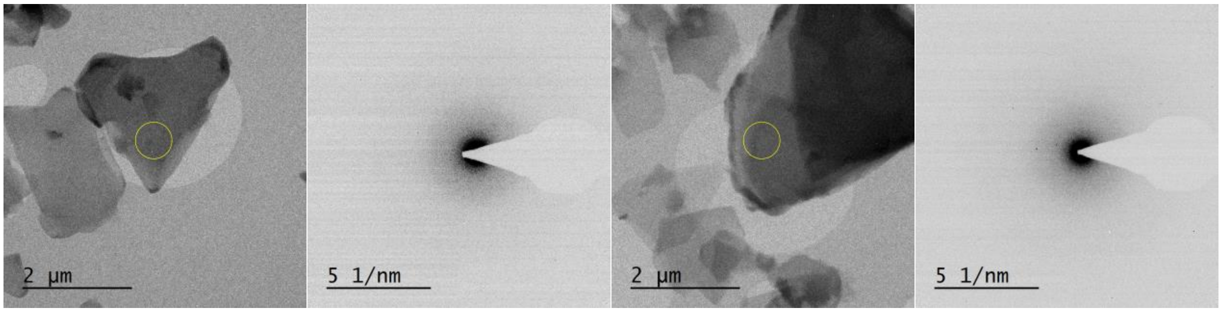

Figure S34: Bright-field TEM images and corresponding SAED patterns (electron dose 0.1 $e^-/Å^2$) of the pyrylium-based 2D polymer **2** after exfoliation in aqueous sodium cholate. The 0-0.4k *g* fraction was used.

In an attempt to clarify this puzzling issue of the loss in crystallinity of the “unexfoliated” fraction of **2**, we subjected the sample to optical extinction and absorbance spectroscopy. The spectra are shown in figure S35. Interestingly, we observe the characteristic signature of the pyrylium-based polymer **1** as a faint peak at 645 nm in the absorbance spectra. In the extinction spectra this feature is mostly masked by non-resonant scattering. Note that this feature was absent in the other size-selected fractions of **2**. We therefore conclude that the post-polymerization of **1** to **2** was not fully

quantitative even though suggested by the other accessible characterization techniques (ss-NMR, IR and XRD).

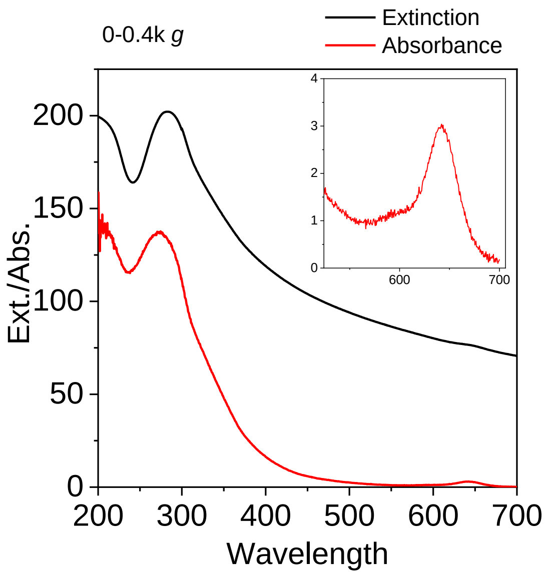

Figure S35: Extinction and absorbance spectrum of the fraction of “unexfoliated” 2DP **2** collected at 0-0.4k g after exfoliation and size selection in aqueous sodium cholate. In the absorbance spectrum, the characteristic feature of 2DP **1** is observed at 645 nm (inset).

Since the charged pyrylium-based polymer **1** cannot be exfoliated as readily in aqueous surfactant as the pyridine-based polymer **2**, **1** will be enriched in the sediment at very low centrifugal acceleration. This accounts for the absence in crystallinity in case of some nanosheets from SAED (figure S34), as we found that **1** undergoes a structural distortion when exfoliated in aqueous sodium cholate (see section 1). This is an important finding, as it illustrates that i) LPE in combination with size selection can lead to a purification of the nanosheets and that ii) an understanding of optical spectra is extremely powerful, as it reveals the presence of minor impurities. From the absorbance coefficients, we estimate that 2-3% of the pyrylium-based polymer **1** were not converted to **2** by the treatment with gaseous ammonia.

V. REFERENCES

[1] R. Z. Lange, G. Hofer, T. Weber, A. D. Schlüter, *J. Am. Chem. Soc.* **2017**, *139*, 2053-2059.

[2] C. Backes, B. M. Szydłowska, A. Harvey, S. Yuan, V. Vega-Mayoral, B. R. Davies, P.-l. Zhao, D. Hanlon, E. J. G. Santos, M. I. Katsnelson, W. J. Blau, C. Gadermaier, J. N. Coleman, *ACS Nano* **2016**, *10* 1589-1601.

[3] *CrysAlis PRO User Manual*, Agilent Technologies Ltd, Yarnton, Oxfordshire, England, **2009**.

[4] R. Herbst-Irmer, G. M. Sheldrick, *Acta Crystallogr., B* **1998**, *54*, 443-449.

[5] O. V. Dolomanov, L. J. Bourhis, R. J. Gildea, J. A. K. Howard, H. Puschmann, *J. Appl. Cryst.* **2009**, *42*, 339-341.

[6] V. Buchholz, V. Enkelmann, *Molecular Crystals and Liquid Crystals Science and Technology. Section A. Molecular Crystals and Liquid Crystals* **1998**, *313*, 309-314.

[7] M. A. Fernandes, D. C. Levendis, *Cryst. Eng. Comm.* **2016**, *18*, 7363-7376.

[8] A. T. Balaban, G. D. Mateescu, M. Elian, *Tetrahedron* **1962**, *18*, 1083-1094.

[9] M. Connolly, *Science* **1983**, *221*, 709-713.

[10] A. Spek, *Acta Crystall. D* **2009**, *65*, 148-155.

[11] aY. Hernandez, M. Lotya, D. Rickard, S. D. Bergin, J. N. Coleman, *Langmuir* **2010**, *26*, 3208-3213; bJ. M. Hughes, D. Aherne, J. N. Coleman, *J. Appl. Polym. Sci.* **2013**, *127*, 4483-4491; cG. Cunningham, M. Lotya, C. S. Cucinotta, S. Sanvito, S. D. Bergin, R. Menzel, M. S. P. Shaffer, J. N. Coleman, *ACS Nano* **2012**, *6*, 3468-3480; dJ. N. Coleman, *Adv. Funct. Mater.* **2009**, *19*, 3680-3695.

[12] J. Lyklema, *Colloids and Surfaces A: Physicochemical and Engineering Aspects* **1999**, *156*, 413-421.

[13] T. Svedberg, K. O. Pederson, J. H. Bauer, *The Ultracentrifuge*, Oxford University Press: London, U.K., **1940**.

[14] aY. Hernandez, V. Nicolosi, M. Lotya, F. M. Blighe, Z. Sun, S. De, I. T. McGovern, B. Holland, M. Byrne, Y. K. Gun'Ko, J. J. Boland, P. Niraj, G. Duesberg, S. Krishnamurthy, R. Goodhue, J. Hutchison, V. Scardaci, A. C. Ferrari, J. N. Coleman, *Nat. Nanotechnol.* **2008**, *3*, 563-568; bJ. N. Coleman, M. Lotya, A. O'Neill, S. D. Bergin, P. J. King, U. Khan, K. Young, A. Gaucher, S. De, R. J. Smith, I. V. Shvets, S. K. Arora, G. Stanton, H.-Y. Kim, K. Lee, G. T. Kim, G. S. Duesberg, T. Hallam, J. J. Boland, J. J. Wang, J. F. Donegan, J. C. Grunlan, G. Moriarty, A. Shmeliov, R. J. Nicholls, J. M. Perkins, E. M. Grieveson, K. Theuwissen, D. W. McComb, P. D. Nellist, V. Nicolosi, *Science* **2011**, *331*, 568-571.

[15] L. Ueberricke, J. N. Coleman, C. Backes, *Phys. Stat. Solidi B* **2017**, *254*, 1700443.

[16] aC. Backes, R. J. Smith, N. McEvoy, N. C. Berner, D. McCloskey, H. C. Nerl, A. O'Neill, P. J. King, T. Higgins, D. Hanlon, N. Scheuschner, J. Maultzsch, L. Houben, G. S. Duesberg, J. F. Donegan, V. Nicolosi, J. N. Coleman, *Nature Commun.* **2014**, *5*, 4576; bD. Hanlon, C. Backes, T. M. Higgins, M. Hughes, A. O'Neill, P. King, N. McEvoy, G. S. Duesberg, B. Mendoza Sanchez, H. Pettersson, V. Nicolosi, J. N. Coleman, *Chem. Mater.* **2014**, *26*, 1751-1763; cA. Harvey, C. Backes, Z. Gholamvand, D. Hanlon, D. McAteer, H. C. Nerl, E. McGuire, A. Seral-Ascaso, Q. M. Ramasse, N. McEvoy, S. Winters, N. C. Berner, D. McCloskey, J. Donegan, G. Duesberg, V. Nicolosi, J. N. Coleman, *Chem. Mater.* **2015**, *27*, 3483–3493; dC. Backes, K. R. Paton, D. Hanlon, S. Yuan, M. I. Katsnelson, J.

Houston, R. J. Smith, D. McCloskey, J. F. Donegan, J. N. Coleman, *Nanoscale* **2016**, *8*, 4311-4323.

[17] aD. Hanlon, C. Backes, E. Doherty, C. S. Cucinotta, N. C. Berner, C. Boland, K. Lee, P. Lynch, Z. Gholamvand, A. Harvey, S. Zhang, K. Wang, G. Moynihan, A. Pokle, Q. M. Ramasse, N. McEvoy, W. J. Blau, J. Wang, G. Abellan, F. Hauke, A. Hirsch, S. Sanvito, D. D. O'Regan, G. S. Duesberg, V. Nicolosi, J. N. Coleman, *Nature Commun.* **2015**, *6*, 8563; bS. Mandeep, G. Enrico Della, A. Taimur, W. Sumeet, R. Rajesh, E. Joel van, M. Edwin, B. Vipul, *2D Mater.* **2017**, *4*, 025110.

[18] L. Yadgarov, C. L. Choi, A. Sedova, A. Cohen, R. Rosentsveig, O. Bar-Elli, D. Oron, H. J. Dai, R. Tenne, *Acs Nano* **2014**, *8*, 3575-3583.

[19] H. C. van de Hulst, *Light Scattering by Small Particles*, Courier Corporation, **1981**.

[20] G. S. He, H.-Y. Qin, Q. Zheng, *J. Appl. Phys.* **2009**, *105*, 023110.
